# Experimental evolution reveals sex‐specific dominance for surviving bacterial infection in laboratory populations of *Drosophila melanogaster*

**DOI:** 10.1002/evl3.259

**Published:** 2021-10-14

**Authors:** Manas Geeta Arun, Amisha Agarwala, Zeeshan Ali Syed, Jigisha ., Mayank Kashyap, Saudamini Venkatesan, Tejinder Singh Chechi, Vanika Gupta, Nagaraj Guru Prasad

**Affiliations:** ^1^ Department of Biological Sciences Indian Institute of Science Education and Research Mohali Mohali 140306 India; ^2^ Department of Biology Syracuse University Syracuse New York 13210; ^3^ Institute of Evolutionary Biology, School of Biological Sciences, King's Buildings University of Edinburgh Edinburgh EH9 3FL United Kingdom; ^4^ Department of Entomology Cornell University Ithaca New York 14853

**Keywords:** Cytogenetic cloning, immunity, immunocompetence, interpopulation crosses, intersexual genetic correlations, sexual conflict, sexual dimorphism, X chromosome, X‐linked variation

## Abstract

Males and females are subjected to distinct kinds of selection pressures, often leading to the evolution of sex‐specific genetic architecture, an example being sex‐specific dominance. Sex‐specific dominance reversals (SSDRs), where alleles at sexually antagonistic loci are at least partially dominant in the sex they benefit, have been documented in Atlantic salmon, rainbow trout, and seed beetles. Another interesting feature of many sexually reproducing organisms is the asymmetric inheritance pattern of X chromosomes, which often leads to distinct evolutionary outcomes on X chromosomes compared to autosomes. Examples include the higher efficacy of sexually concordant selection on X chromosomes, and X chromosomes being more conducive to the maintenance of sexually antagonistic polymorphisms under certain conditions. Immunocompetence is a trait that has been extensively investigated for sexual dimorphism with growing evidence for sex‐specific or sexually antagonistic variation. X chromosomes have been shown to harbor substantial immunity‐related genetic variation in the fruit fly, *Drosophila melanogaster*. Here, using interpopulation crosses and cytogenetic cloning, we investigated sex‐specific dominance and the role of the X chromosome in improved postinfection survivorship of laboratory populations of *D. melanogaster* selected against pathogenic challenge by *Pseudomonas entomophila*. We could not detect any contribution of the X chromosome to the evolved immunocompetence of our selected populations, as well as to within‐population variation in immunocompetence. However, we found strong evidence of sex‐specific dominance related to surviving bacterial infection. Our results indicate that alleles that confer a survival advantage to the selected populations are, on average, partially dominant in females but partially recessive in males. This could also imply an SSDR for overall fitness, given the putative evidence for sexually antagonistic selection affecting immunocompetence in *Drosophila melanogaster*. We also highlight sex‐specific dominance as a potential mechanism of sex differences in immunocompetence, with population‐level sex differences primarily driven by sex differences in heterozygotes.

## Introduction

Males and females experience distinct selection pressures (Bonduriansky and Chenoweth 2009) often resulting in sex‐specific genetic architectures (Lande 1980). For example, the dominance coefficients of alleles can be different between the sexes. An interesting special case of this is sex‐specific dominance reversal (SSDR) where, in conjunction with sexually antagonistic (SA) selection, alleles that benefit females (males) are at least partially dominant in females (males) (Fry 2010; Connallon and Chenoweth 2019). Mirroring the debate between R. A. Fisher and Sewall Wright surrounding the evolution of dominance in general (Provine 1992; Otto and Bourguet 1999), there are two proposed mechanisms for SSDRs at SA loci. Like Wright, Fry (2010) argued that SSDRs at SA loci could be a by‐product of concavity of fitness surfaces near fitness optima, with heterozygotes of a given sex having fitness closer to the fitter homozygote of that sex. On the other hand, Spencer and Priest (2016), like Fisher, showed that selection could favor the evolution of modifier alleles that modulate dominance coefficients at SA loci in a sex‐dependent manner. Evidence for SSDR is minimal, but includes age of maturation in salmon (Barson et al. 2015), sex‐specific migratory patterns in rainbow trout (Pearse et al. 2019), and overall fitness in seed beetles (Grieshop and Arnqvist 2018).

Another interesting feature of many sexually reproducing organisms is the asymmetric inheritance patterns of sex chromosomes, which cause the outcomes of several evolutionary processes to be different between sex chromosomes and autosomes. For example, in male heterogametic systems, sexually concordant positive (Charlesworth et al. 1987; Meisel and Connallon 2013) as well as purifying (Vicoso and Charlesworth 2006; Avery 1984) selection is predicted to be more efficient on the X chromosome. Consequences of SA selection, too, can be distinct for X chromosomes and autosomes, with X chromosomes predicted to be more efficient at maintaining SA polymorphisms when female beneficial alleles are at least partially dominant in both sexes (Rice 1984), but not necessarily in general (Pamilo 1979; Curtsinger 1980; Patten and Haig 2009; Fry 2010; Connallon and Clark 2012). Nevertheless, several studies have detected appreciable X‐linked SA variation in insects (Gibson et al., 2002; Pischedda and Chippindale 2006; Fedorka and Mousseau 2004; Long et al. 2012; Ruzicka et al. 2019), reptiles (Calsbeek & Sinervo 2004), and mammals (Foerster et al. 2007; Lucotte et al. 2016) (but see Ruzicka and Connallon 2020). SA selection can lead to the evolution of sex‐biased gene expression (Connallon and Clark 2010), leading to sexual dimorphism (Lande 1980). Several studies have investigated the genomic distribution (autosomes vs. X chromosomes) of sex‐biased genes with mixed results (summarized in Jaquiéry et al. 2013; Dean and Mank 2014). There is, however, evidence for X‐linked sex‐dependence or dimorphism in traits such as body size (Mathews et al. 2017), locomotory activity (Long and Rice 2007), and life span (Griffin et al. 2016). Sex chromosomes have also been predicted to generate sex differences in trait variation (Connallon 2010), with the heterogametic sex exhibiting more trait variation than their homogametic counterpart, a pattern reported by multiple empirical studies (Reinhold and Engqvist 2013; Wyman and Rowe 2014; Griffin et al. 2016).

Immunocompetence is an ideal trait to investigate sex‐specific effects. Females generally exhibit superior immunocompetence relative to males in vertebrates (Poulin 1996; Zuk and McKean 1996) and invertebrates (Nunn et al. 2009; Kelly et al. 2018 but see Sheridan et al. 2000). Several evolutionary explanations for these patterns, such as the immunocompetence handicap hypothesis (Folstad and Karter 1992), trade‐offs between male immunocompetence and ornamentation (Sheldon and Verhulst 1996), the Bateman principle (Rolff 2002) (but see Stoehr and Kokko 2006), and interactions between immune trade‐offs and reproductive schedules (Metcalf and Graham 2018) indicate sex‐specific fitness optima, and therefore, a potential for SA selection (Rolff 2002; Roved et al. 2017). Consistent with this idea, negative intersexual fitness covariances over immunocompetence‐related traits have been reported in side‐blotched lizards (Svensson et al. 2009), seed beetles (Bagchi et al. 2021), and fruit flies (Vincent and Sharp 2014; Sharp and Vincent 2015; Hill‐Burns and Clark 2009). However, given the complex relationship between immunocompetence and male reproductive fitness (Stoehr and Kokko 2006), whether these patterns imply sexual antagonism for fitness remains unclear. There is also comprehensive genetic evidence for sex dependence for disease‐related traits in humans (Gilks et al. 2014; Khramtsova et al. 2019).

*Drosophila melanogaster* is an ideal model to investigate sex specificity in immunocompetence. *D. melanogaster* immune function has been widely studied from a mechanistic point of view (Hoffmann 2003; Leclerc and Reichhart 2004; Buchon et al. 2014) with many immunocompetence‐related genes located on the X chromosome (Hill‐Burns and Clark, 2009). There is evidence for sexual dimorphism (reviewed in Belmonte et al. 2020) as well as sexual antagonism for immunocompetence in *D. melanogaster* (Vincent and Sharp, 2014; Sharp and Vincent 2015). It is not clear, however, whether this sexual antagonism is due to X‐linked loci or is associated with sex‐specific dominance. Also, *D. melanogaster* X chromosomes are large (nearly 20% of the total genome) (Turelli and Begun 1997). Therefore, the power to detect X‐linked effects is maximized. Hill‐Burns and Clark (2009) detected considerable X‐linked variation for bacterial clearance ability in *D. melanogaster*, which was partly sexually dimorphic or antagonistic. However, bacterial clearance ability may not necessarily correlate with fitness upon infection by bacteria. For example, an organism could maintain fitness post infection, by deploying increased tolerance (which does not decrease pathogen fitness [Vincent and Sharp 2014; Sharp and Vincent 2015]) as opposed to resistance. Therefore, to the best of our knowledge, the role played by X chromosomes in facilitating adaptation to pathogenic challenge, as well as dominance effects associated with immunocompetence, has not yet been comprehensively investigated.

In the present study, we used replicate laboratory populations of *D. melanogaster* selected against infection by *Pseudomonas entomophila* and their respective controls (Gupta 2016; Gupta et al. 2016) to address the following questions:

1. Was there sex‐specific dominance for surviving *P. entomophila* infection associated with alleles that increased in frequency in the selected populations?
2. Was there measurable X‐linked variation for surviving *P. entomophila* infection in the selected and control populations? If yes, was this variation comparable between the sexes?
3. Were evolved survivorship differences between selected and control populations, at least partly, due to evolved differences between X chromosomes from selected and control populations?

*Pseudomonas entomophila* is a gram‐negative bacterium isolated from a wild *D. melanogaster* female (Dieppois et al. 2015). Infection by *P. entomophila* is lethal to *D. melanogaster*; it causes a certain fraction of infected individuals to die depending on the dosage. Therefore, it has been used in studies investigating evolution of *D. melanogaster* immunocompetence in face of bacterial infection (Martins et al. 2013; Faria et al. 2015).

To address our questions, first, we performed reciprocal crosses between selected and control populations (à la Hoffmann and Parsons 1989; Joshi et al. 1996; Vijendravarma and Kawecki 2013, 2015), and measured the postinfection survivorship of the F1 progeny. Male progeny from the two hybrid crosses inherited X chromosomes from either the selected or control populations, but had comparable autosomes. We also used these data to estimate sex‐specific dominance for surviving bacterial infection. Second, using cytogenetic cloning (Gibson et al. 2002) we sampled 80 X chromosomes each from selected and control regimes, and expressed them in males and females carrying the rest of the genome from the ancestral baseline population. The immunocompetence of these flies was assayed by measuring their survivorship post infection. 

## Methods

### FLY POPULATIONS

Below, we present a summary of our experimental populations (Wolbachia infection status unknown). The detailed maintenance protocols can be found in Section A in the Supporting Information.

### ANCESTRAL POPULATIONS

The I and S populations (see below) used in our experiments were derived from four large (∼2800 individuals each), laboratory populations of *D. melanogaster* called Blue‐Ridge Baselines (BRB_1‐4_). BRBs are maintained on a 14‐day discrete generation cycle, at 25°C, on banana‐jaggery‐yeast medium, on a 12 h:12 h light:dark cycle.

### SELECTION REGIMES

Both I and S regimes consisted of four replicate populations (Gupta 2016; Gupta et al. 2016). For each population in the I regime (I_1‐4_), 150 males and 150 females were infected (see Section A in the Supporting Information for protocol) with *Pseudomonas entomophila* strain L48 every generation, leading to ∼33% mortality over 96 h post infection. For each population in the S regime (S_1‐4_), every generation, 100 males and 100 females were “sham‐infected” with sterile MgSO_4_, which does not cause mortality. To start the next generation, eggs laid in the 18‐h window starting 96 h post infection/sham‐infection were collected at a density of 70 eggs per vial, each containing 8‐ to 10‐ml food. Each population consisted of 10 such vials. Note that each of the four BRB populations were used to derive one I and one S population (I_1_ and S_1_ were derived from BRB_1_, and so on). Pairs of I and S populations sharing a common subscript were also handled together during regular maintenance and experiments. Therefore, they form four statistical “blocks.”

### FLY STOCKS FOR CYTOGENETIC CLONING

Clone Generator (CG) females (Rice 1996) have a compound X chromosome and a homozygous‐viable translocation between the two major autosomes. DxBRB females have a compound X chromosome, and autosomes derived from the BRB_1_ populations.

We performed two distinct assays, as summarized below. For details, refer to Section A in the Supporting Information.

#### Hybrid Experiment

We performed this experiment between generations 65 and 75 of selection. For each block, we collected virgin males and females from I and S populations and crossed them with each other to set up the following crosses (100 pairs per cross):

1. I ♀ ×  I ♂ (II)
2. S ♀ ×  S ♂ (SS)
3. I ♀ ×  S ♂ (IS)
4. S ♀ ×  I ♂ (SI)

For each cross, we assayed the survivorship of male and female F1 offspring post infection by *P. entomophila* (OD_600_ = 1.5). In every block, for each cross, we set up three cages (14 cm × 16 cm × 13 cm), each containing 50 infected females and 50 infected males, and a cage containing 50 males and 50 females that were “sham‐infected” as injury controls. Mortality was recorded for 96 h post infection. Male progeny from IS and SI crosses inherited their X chromosomes from I and S females, respectively, but had similar autosomes. Therefore, survivorship differences between male offspring from IS and SI crosses could be attributed to X‐linked loci. However, IS and SI males also inherited Y chromosomes (mitochondria) from S (I) and I (S) regimes, respectively. To rule out a possible confounding effect of the Y chromosome or mitochondria (see *Discussion*), we performed the X‐Cloning Experiment.

#### X‐Cloning Experiment

After 160 generations of selection, using cytogenetic cloning techniques (Gibson et al. 2002), we randomly sampled 20 X chromosomes from each of the four I and S population, each used to create a single X‐line. Thus, we had a total of 80 X‐lines from the I regime and 80 X‐lines from the S regime. In a series of four crosses involving the CG females, DxBRB females and BRB_1_ females (see Section A in the Supporting Information, Fig. S1), we expressed these X chromosomes in males and females carrying the rest of the genome sampled randomly from the ancestral BRB_1_ population. Note that these females were heterozygous for the target X chromosome, with the other X chromosome being from the BRB_1_ population. Subsequently, we assayed the survivorship of these flies post infection by *P. entomophila* (OD_600_ = 1.0). For each X‐line, per sex, we set up three food vials (90 mm length × 2.5 mm diameter) containing eight infected individuals each and one vial containing eight sham‐infected individuals (see Section A in the Supporting Information). Mortality was recorded for 96 h post infection.

### STATISTICAL ANALYSIS

There was negligible mortality in the sham (i.e., injury‐control) treatment. Therefore, data for the sham treatment were excluded. All analyses were performed in R (version 4.0.2) (R Core Team 2018). Below, we briefly outline our analyses; the details are described in Section B in the Supporting Information.

#### Hybrid Experiment

We used the packages “lme4” (Bates et al. 2015) and “lmerTest” (Kuznetsova et al. 2017) to fit a linear mixed‐effects model for proportion survivorship, and a logistic regression on the status of the flies at 96 h post infection (dead or alive). Using the package “coxme” (Therneau 2020), we fit a Cox proportional hazards model. In these analyses, cross and sex were fixed factors, whereas blocks were random.

**Figure 1:**
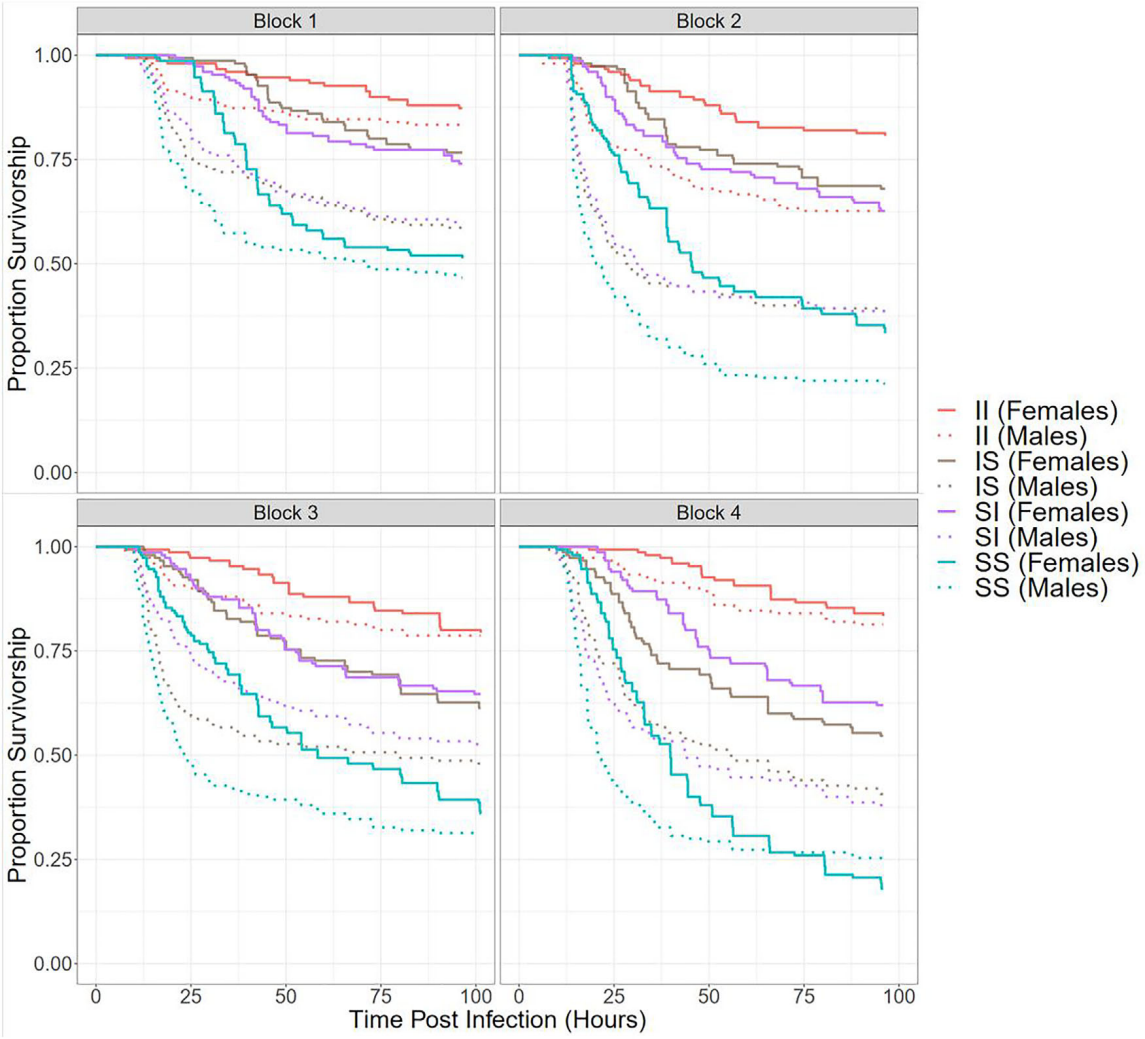
Effect of cross (II, red; IS, brown; SI, purple; SS, blue) and sex (solid, females; dotted, males) on survivorship post infection in the Hybrid Experiment. The curves show survival of the F1 progeny as a function of time. The first letter indicates the maternal selection regime and the second, the paternal.

Because our results suggested that in terms of survivorship post infection, the hybrid crosses (IS and SI) were closer to the II cross in females but the SS cross in males (Fig. 1), we investigated the possibility of sex‐specific dominance. Dominance coefficient (see Section B in the Supporting Information) for proportion survivorship was calculated separately for males and females for each of the four blocks, as well as for the entire dataset together. We used a stratified bootstrap approach using the R package “boot” (Canty and Ripley 2020) to construct 95% confidence intervals (CIs) around our point estimates of dominance coefficients for males (D_m_) and females (D_f_). We also constructed 95% CIs for the difference in the dominance coefficients for females and males (D_f_ – D_m_) and examined if they included 0.

#### X‐Cloning Experiment

We used “lme4” and “lmerTest” to fit linear mixed‐effects models for proportion survivorship and median time to death, and a logistic regression on the status of the flies (dead or alive) at 96 h post infection. We also fit a Cox proportional hazards model using “coxme.” Selection regime and sex were modeled as fixed factors, whereas block and X‐line were random. To formally investigate X‐linked variation for postinfection survivorship in I and S selection regimes, we used the package “MCMCglmm” (Hadfield 2010) to fit a Bayesian mixed model for median time to death. We calculated estimates of male and female heritabilities associated with the X chromosome as well as X‐linked intersexual genetic correlation for median time to death. We further calculated the difference between male and female heritability and the corresponding 95% credible intervals in each selection regime to test the potential for differential X‐linked heritability of postinfection survivorship between the sexes. To test for the effect of X‐line, we fit a null model that lacked a term corresponding to X‐line and compared its Deviance Information Criterion (DIC) to that of the model incorporating X‐line.

Additionally, we also investigated male‐female correlations among X‐lines (using Spearman's rank correlation and linear models) for average median time to death and proportion survivorship, separately for each of the eight experimental populations (I_1‐4_ and S_1‐4_). 

## Results

### HYBRID EXPERIMENT

In our linear mixed‐effects model, we found a significant effect of sex (*F*_1,3_ = 14.3406, *P* = 0.0314), cross (*F*_3,80_ = 67.851, *P* < 0.0001), and the interaction between the two (*F*_3,80_ = 3.0358, *P* = 0.0339) (Table 1, panel A). Females had higher survivorship overall. In both sexes, the II cross had the highest survivorship, followed by IS and SI, which were not significantly different (Tukey's HSD, females: *P* > 0.9999, males: *P* > 0.9999) from each other. SS had the lowest survivorship (Figs. 1, S2A, B, and S3; Table S1). The effect of sex was primarily a result of sex differences within the hybrid crosses (i.e., IS or SI flies) (Figs. 1, S2A, B, and S3). In the II (Tukey's HSD, *P* = 0.9157) and SS (Tukey's HSD, *P* = 0.9962) crosses, males and females were not significantly different, but in the hybrid crosses (IS and SI), males fared significantly worse than females (Tukey's HSD, IS: *P* = 0.026, SI: *P* = 0.026; Table S1). The results of our Cox proportional hazards model and logistic regression were qualitatively similar (Table 1, panels B and C; Table S2). 

**Table 1:** Summary of Hybrid Experiment results. (A) Linear mixed‐effects model of proportion survivorship. (B) Cox proportional hazards model of survivorship post infection. (C) Logistic regression. Note that for the Cox proportional hazard model and logistic regression, coefficients for fixed factors are calculated relative to the default level (which is constrained to be 0) for that fixed factor. The default level for cross is “II” and the default level for sex is “female.”

**Hybrid Experiment**
**(A) Proportion survivorship**

	Sum Sq	Mean Sq	NumDF	DenDF	*F*‐value	*P*‐value
Cross	2.6139	0.8713	3	80	67.851	<0.0001
Sex	0.1880	0.1880	1	3	14.6406	0.0314
Cross:Sex	0.1170	0.0390	3	80	3.0358	0.0339

	npar	logLik	AIC	LRT	Df	*P*‐value
<none>	13	49.098	–72.196			
(1 | Block)	12	47.932	–71.865	2.3313	1	0.1268
(1 | Sex:Block)	12	48.82	–73.639	0.5570	1	0.4555
(1 | Cross:Block)	12	49.098	–74.196	0.0000	1	>0.9999
(1 | Infector)	12	33.666	–43.332	30.8639	1	<0.0001
**(B) Cox proportional hazards**						

Fixed coefficients	coef	se(coef)	*z*‐value	*P*‐value
CrossIS	0.8241	0.1209	6.82	<0.0001		
CrossSI	0.7901	0.1213	6.51	<0.0001		
CrossSS	1.7038	0.1115	15.28	<0.0001		
SexMale	0.4146	0.1508	2.75	0.0060		
CrossIS:SexMale	0.3857	0.1583	2.44	0.0150		
CrossSI:SexMale	0.3650	0.1587	2.3	0.0210		
CrossSS:SexMale	–0.0073	0.1487	–0.05	0.9600		

Random effects	Variance					
Infector	0.1843					
Block/Sex/Cross	0.0004					
Block/Sex	0.0115					
Block	0.0951					
**(C) Logistic regression**						

Fixed effects	Estimate	Std. Error	*z*‐value	*P*‐value		
(Intercept)	1.6427	0.3248	5.058	<0.0001		
CrossIS	–0.9751	0.1636	–5.959	<0.0001		
CrossSI	–0.9449	0.1638	–5.768	<0.0001		
CrossSS	–2.3197	0.1646	–14.094	<0.0001		
SexMale	–0.3964	0.1471	–2.694	0.0071		
CrossIS:SexMale	–0.4173	0.1912	–2.183	0.0290		
CrossSI:SexMale	–0.4194	0.1913	–2.192	0.0284		
CrossSS:SexMale	0.2266	0.1945	1.1650	0.2439		

Random effects	Variance					
Block:Cross	0.01383					
Block	0.12483					
Infector	0.1761					

**Figure 2:**
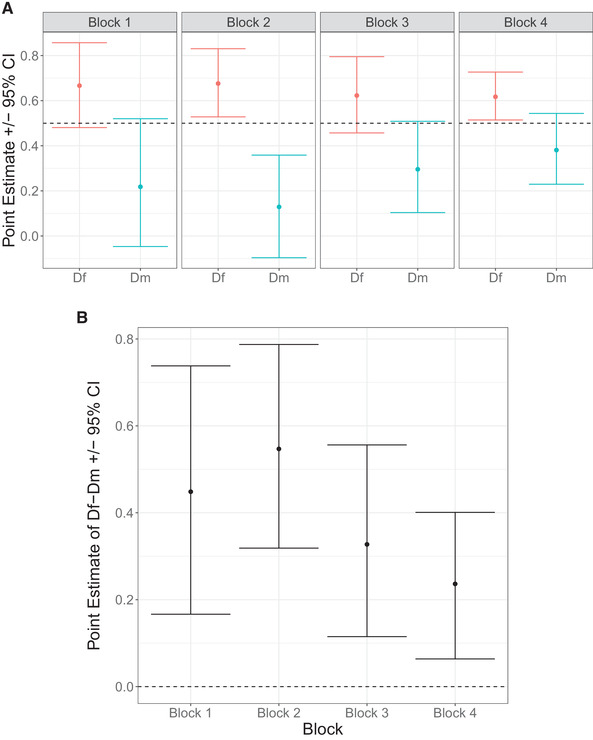
(A) Point estimates and 95% confidence intervals for dominance coefficients for proportion survivorship in females (D_f_) and males (D_m_) for each of the four blocks. Confidence intervals were generated by bootstrapping the dataset 10,000 times (see Statistical Analysis). The horizontal dashed line corresponds to complete additivity (i.e., dominance coefficient = 0.5). (B) Point estimates and 95% confidence intervals for the difference in the dominance coefficients for proportion survivorship in females and males (i.e., D_f_ – D_m_) for each of the four blocks. Confidence intervals were generated by bootstrapping the dataset 10,000 times (see Statistical Analysis). The horizontal dashed line corresponds to dominance coefficients being equal between the sexes (i.e., D_f_ – D_m_ = 0).

When data from all blocks were combined, the dominance coefficient for females was significantly greater than 0.5 [*D*_f_ = 0.6424, 95% CI = (0.5702, 0.7140)], whereas the dominance coefficient for males was significantly smaller than 0.5 [D_m_ = 0.2684, 95% CI = (0.1689, 0.3760)] (Fig. S4). The difference between the dominance coefficients of females and males was significantly greater than 0 [D_f_ – D_m_ = 0.3740, 95% CI = (0.2693, 0.4835)] (Fig. S4). The results when blocks were analyzed separately were largely consistent with this. In each block, the 95% CIs for D_f_ – D_m_ did not overlap with 0 (Fig. 2B). In each block, D_f_ was greater than 0.5, although this trend was statistically significant only in block 2 and block 4. In contrast, in every block, D_m_ was smaller than 0.5, but this trend was statistically significant only in block 2 (Fig. 2A).

### X‐CLONING EXPERIMENT

The linear mixed‐effects model for proportion survivorship suggested a significant effect of sex (*F*_1,158.01_ = 12.6633, *P* = 0.0005), with males having marginally higher survivorships than females, whereas there was no effect of selection regime (*F*_1, 155.07_ = 1.4284, *P* = 0.2338), or its interaction with sex (*F*_1,158.01_ = 0.0423, *P* = 0.8374) (Table 2, panels A and C; Table S3, panel B; Fig. S5A, B). The output of the logistic regression was qualitatively similar. The Cox proportional hazards model (Table 2, panel D; Table S3, panel A; Figs. 2 and S6) and the linear mixed‐effects model for median time to death (Table 2, panel B; Fig. S7A, B) failed to detect any effect of selection regime (*F*_1,156.13_ = 1.6122, *P* = 0.2061), sex (*F*_1,801.11_ = 0.0406, *P* = 0.8403), or their interaction (*F*_1,801.11_ = 0.0014, *P* = 0.9699). Additionally, neither of our linear mixed‐effects models detected an effect of the X‐line (Likelihood ratio test, median time to death: *P* = 0.6718, proportion survivorship: *P* = 0.9572) (Table 2, panels A and B). Our Bayesian analyses could also not detect a significant effect of X‐line in both the selection regimes as null models that lacked a term corresponding to X‐line had lower DIC (1342.306 and 1351.837 for I and S, respectively) than the corresponding models incorporating X‐line (1344.905 and 1354.273 for I and S, respectively) (Table S4). The mean X‐linked heritabilities for median time to death and the corresponding 95% credible intervals (CIs) for I females, I males, S females, and S males were 0.0224 (CI = [5.9678 × 10^−9^, 0.0845]), 0.0262 (CI = [9.1931 × 10^−10^, 0.0890]), 0.0450 (CI = [5.2707 × 10^−9^, 0.1525]), and 0.0168 (CI = [1.4747 × 10^−8^, 0.0622]), respectively. Their posterior distributions (constrained to be positive in the models) had a sharp peak in the neighborhood of 0 (Fig. S8A–D) and decayed rapidly further away. The 95% CIs for the difference between male and female heritability, for I regime (−0.0966, 0.0945) and S (−0.1514, 0.0732) regime, overlapped with 0 (Fig. S8E, F). The X‐linked intersexual genetic correlation for median time to death was 0.0825 [CI = (−0.8032, 0.9048)] for the I regime and 0.1074 [CI = (−0.7516, 0.9444)] for the S regime.

**Table 2:** Summary of X‐Cloning Experiment results. (A) Logistic regression, linear mixed‐effects model of (B) median time to death and (C) proportion survivorship, and (D) Cox proportional hazards model of survivorship post infection. Note that for the Cox proportional hazard model and the logistic regression, coefficients for fixed factors are calculated relative to the default level (which is constrained to be 0) for that fixed factor. The default level for selection is “I” and the default level for sex is “female.”

**X‐Cloning Experiment**
**(A) Logistic regression**

Fixed effects	Estimate	Std. Error	*z*‐value	*P*‐value		
(Intercept)	–3.2541	0.2689	–12.1	<0.0001		
SelectionS	–0.2443	0.1857	–1.315	0.1884		
Sexmale	0.4386	0.1401	3.13	0.0018		
SelectionS:Sexmale	0.1502	0.2069	0.726	0.4678		

Random effects	Variance					
Xline:Block:Selection	0.3050					
Block	0.0566					
Infector	0.1235					
**(B) Median time to death**						

	Sum Sq	Mean Sq	NumDF	DenDF	*F*‐value‐	*P*‐value
Selection	439354	439354	1	156.13	1.6122	0.2061
Sex	11070	11070	1	801.11	0.0406	0.8403
Selection:Sex	387	387	1	801.11	0.0014	0.9699

	npar	logLik	AIC	LRT	Df	*P*‐value
<none>	8	–7360.8	14738			
(1 | Infector)	7	–7374.8	14764	28.0106	1	<0.0001
(1 | Block)	7	–7373.9	14762	26.2017	1	<0.0001
(1 |Xline:Selection:Block)	7	–7360.9	14736	0.1795	1	0.6718
**(C) Proportion survivorship**						

	Sum Sq	Mean Sq	NumDF	DenDF	*F*‐value	*P*‐value
Selection	0.0054	0.0054	1	155.07	1.4284	0.2338
Sex	0.0476	0.0476	1	158.01	12.6633	0.0005
Selection:Sex	0.0002	0.0002	1	158.01	0.0423	0.8374

	npar	logLik	AIC	LRT	Df	*P*‐value
<none>	7	422.33	–830.66			
(1| Xline:Block:Selection)	6	422.33	–832.66	0.0029	1	0.9572
(1 | Block)	6	419.92	–827.84	4.8251	1	0.0281
**(D) Cox proportional hazards**						

Fixed coefficients	coef	se(coef)	*z*‐value	*P*‐value
SelectionS	0.0583	0.0558	1.05	0.3000		
Sexmale	0.0131	0.0342	0.38	0.7000		
SelectionS:Sexmale	0.0293	0.0483	0.61	0.5400		

Random effects	Variance			
Block/Selection/Xline	0.0777					
Block/Selection	<0.0001					
Block	0.0827					
Infector	0.0368					

For both median time to death and proportion survivorship, there were no significant male‐female correlations in any of the populations (Figs. S9A, B, S10, and S11; Table S5, panels A and B) with the exception of I2, in which there was a positive but weak (*R* ^2^ = 0.353) correlation.

Thus, we could detect neither differences between X chromosomes sampled from I and S regimes nor any within‐population X‐linked variation for survivorship post infection.

## Discussion

Results from the Hybrid Experiment show that flies from the two hybrid crosses (IS and SI) had comparable postinfection survivorships. However, sex differences in survivorship were much more prominent in the hybrid crosses (IS and SI) than in the two parental crosses (II and SS). Consequently, survivorship of the hybrid crosses was closer to II in females, but closer to SS in males, suggesting sex‐specific dominance for surviving infection by *P. entomophila*. Because males from IS and SI crosses inherited their X chromosomes from I and S regimes, respectively, but had comparable autosomes, this suggests that (a) survivorship differences between I and S populations are unlikely to be due to the X chromosome, and (b) the sex‐specific dominance effect cannot be caused by the X chromosome, and is likely to be autosomal in origin. Consistent with this interpretation, we could not distinguish between X chromosomes sampled from I and S populations in the X‐Cloning Experiment. We could also not detect a significant effect of X‐line in any of our analyses, or any significant male‐female correlations (with one exception). Male and female X‐linked heritabilities were small, and not significantly different from each other. Below, we discuss the two key findings of our experiments.

### SEX‐SPECIFIC DOMINANCE

In females, the survivorship of the hybrid crosses (IS and SI) was significantly higher than expected under complete additivity (i.e., midway between II and SS), whereas for males it was significantly lower than the midway point between II and SS. Furthermore, the difference in the dominance coefficients in females and males was significantly different from zero, providing clear evidence of sex‐specific dominance for surviving bacterial infection. Although sex‐specific dominance has been reported for age of maturation (Barson et al. 2015), dispersal patterns (Pearse et al. 2019), and reproductive fitness (Grieshop and Arnqvist 2018), this is among the first reports of sex‐specific dominance for any immunocompetence‐related trait. 

SSDRs can greatly broaden the parameter space for which SA selection can maintain balanced polymorphisms (Kidwell et al. 1977; Fry 2010; Connallon and Clark 2011, 2012). SSDRs can arise through two distinct mechanisms, either as a by‐product of concavity of fitness landscapes and SA selection (see fig. 2 of Fry 2010) or through the evolution of modifier alleles that modulate dominance coefficients at SA loci in a sex‐specific manner (Spencer and Priest 2016). Although our results cannot distinguish between these two alternatives, it is likely that sex‐specific dominance for immunocompetence in our populations is a relic of SA selection in the wild ancestors of our populations. During our laboratory selection experiment, there was strong sexually concordant selection for improved postinfection survivorship. However, it is conceivable that in the wild, alleles that conferred a postinfection survival advantage were favored in females, whereas “immunodeficient” alleles were favored in males through their pleiotropic action on male fitness in other contexts (e.g., reproduction) (Rolff 2002; McKean and Nunney 2005). Studies have shown that I males have poorer mating success when directly competing with S males (Venkatesan 2015). This SA selection could have resulted in sex‐specific dominance for immunocompetence such that female‐beneficial “higher survivorship” alleles were partially dominant in females, but partially recessive in males. During the course of our laboratory selection, alleles that conferred a postinfection survival advantage to both sexes, but were more dominant in females than in males, increased in frequency in the I populations as a result of strong directional selection imposed by the pathogen. It must be noted, however, this assumes that there is a linear relationship between postinfection survivorship and overall fitness. For females, the probability of surviving infection is a reasonable measure of fitness. However, for males, the relationship between survival and fitness is less clear (Stoehr and Kokko 2006). Therefore, although our results provide strong evidence of sex‐specific dominance for surviving bacterial infection, a better understanding of immunocompetence and total fitness is required to determine if these patterns contribute toward maintaining SA polymorphisms.

Nevertheless, we show that, sex differences in immunocompetence at a population level could arise solely through the difference in the immunocompetence of heterozygous males and females. This would, of course, require the maintenance of heterozygotes at sufficiently high frequencies, through processes such as trade‐offs between male immunocompetence and reproductive output. Thus, our results provide a useful insight into the evolution of sex differences in immunocompetence.

### NO EVIDENCE OF AN EFFECT OF X CHROMOSOME ON SURVIVORSHIP POST INFECTION

Our results show that male progeny from IS and SI crosses (which had comparable autosomes but carried X chromosomes from I or S regimes, respectively) had indistinguishable postinfection survivorships. This suggests that X chromosomes from I and S populations were similar in their contribution to this trait, at least in males. IS and SI males also differed in the ancestry (I vs. S) of their Y chromosomes as well as mitochondria. Although *D. melanogaster* Y chromosomes (Kutch and Fedorka 2015, 2017; also see Lund‐Hansen et al. [2021] for X‐Y interactions in *D. melanogaster*) and mitochondria (Salminen and Vale 2020) have been shown to influence immunocompetence, it is unlikely that our findings were confounded by an effect of the Y chromosome or the mitochondria. In the X‐Cloning Experiment, where all the Y chromosomes and mitochondria from I and S regimes were replaced by ancestral Y chromosomes and mitochondria, respectively (Fig. S1), we could not distinguish between X‐lines from I and S regimes. Additionally, in the Hybrid Experiment, IS and SI females, which had comparable chromosomes, but inherited their mitochondria from I and S females, respectively, had indistinguishable survivorships (Table S1), suggesting that I and S mitochondria were comparable in their contribution to survivorship post infection. This clearly suggests that the improvement in the immunocompetence of the I populations did not involve the X chromosome. In contrast to the idea that the heterogametic sex should exhibit greater trait variability (Connallon 2010; Reinhold and Engqvist 2013), our results suggest that males and females had comparable X‐linked heritabilities for surviving bacterial infection. Additionally, in none of our analyses could we detect significant intrapopulation X‐linked variation or intersexual correlations for surviving the infection (with one exception). 

Although a lack of X‐linked variation in the I regime is unsurprising due to strong directional selection for surviving bacterial infection, a lack of any effect of X‐line in the S regime is interesting. It is important to note that the ancestral BRB populations have ample genetic variation for immunocompetence (Gupta 2016), highlighted further by the rapid response to selection reported in I populations (Gupta et al. 2016). Therefore, this apparent (a) lack of X‐linked variation within I and S populations, and (b) absence of evolved differences between X chromosomes from I and S regimes, is remarkable, given that the *D. melanogaster* X chromosome contains 20% of the total genome (Turelli and Begun 1997).

**Figure 3:**
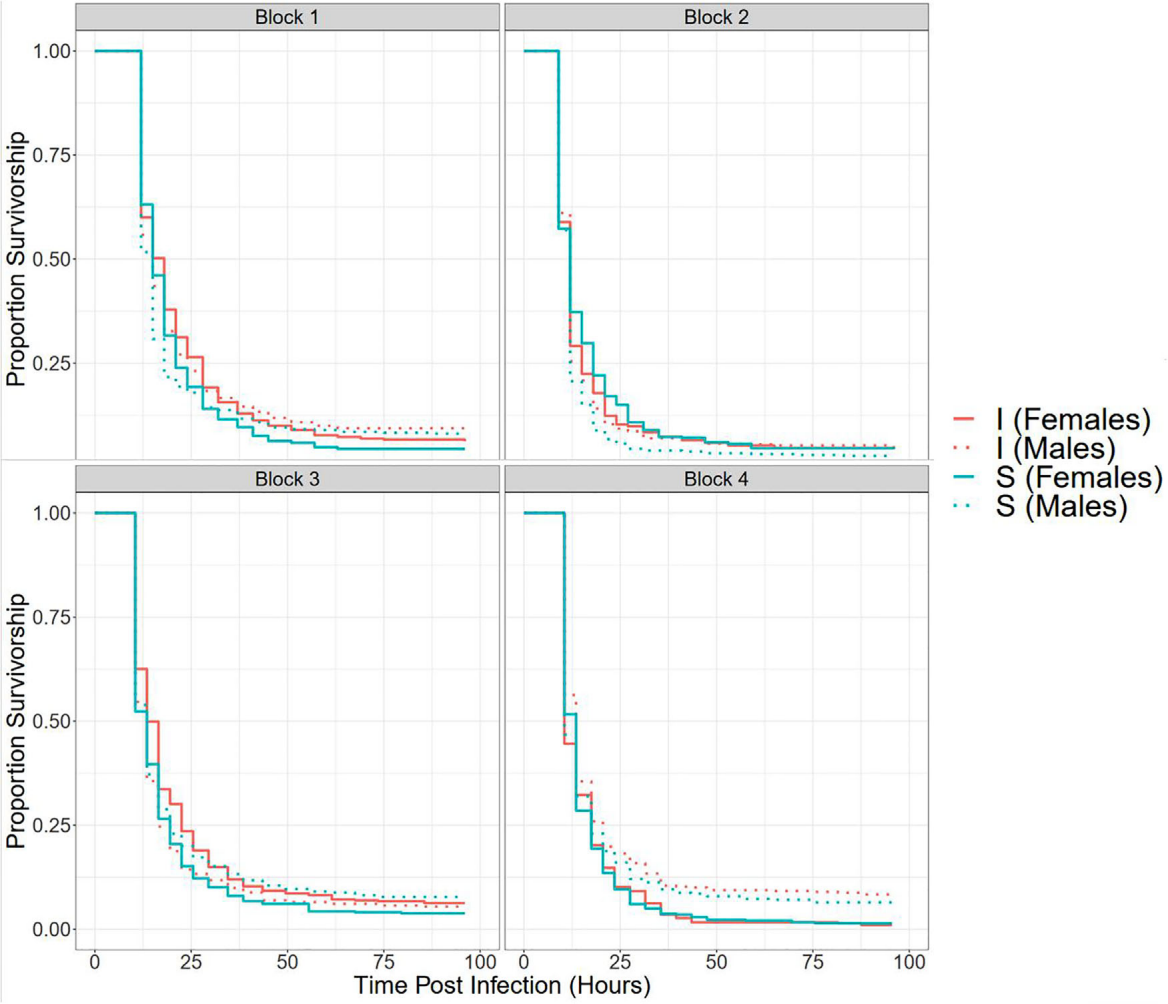
Effect of selection regime (red, I; blue, S) and sex (solid, females; dotted, males) on survivorship post infection in the X‐Cloning Experiment. I and S flies carry the X chromosome from the respective selection regime but share the rest of the genome, which comes from a baseline population.

Our results are in stark contrast to the findings of Hill‐Burns and Clark (2009), who had reported considerable immunity‐related variation on the X chromosome. However, Hill‐Burns and Clark (2009) had used bacterial clearance ability as a measure of immunocompetence, which may not necessarily translate to improved survival in face of pathogenic infection. Additionally, they had used an X chromosome balancer line with inbred autosomes to express target X chromosomes in an isogenic autosomal background in hemizygous state in males and homozygous state in females. On the other hand, because we were also interested in *evolved differences* on the X chromosome between selection regimes, we measured the performance of target X chromosomes averaged over a large number of autosomal backgrounds sampled from the ancestral population. By using diverse “local” autosomal backgrounds similar to the ones the target X chromosomes had evolved with during laboratory selection, we avoided possible spurious epistatic interactions between X chromosomes and a specific “foreign” autosomal background, which could have masked evolved differences. A drawback of this approach was the X‐Cloning Experiment had low power to detect *within‐population* X‐linked effects, because uncontrolled autosomal variation among X‐lines could have masked X‐linked variation. However, this is unlikely to affect our interpretation, because both of our experiments were sufficiently powered to detect *interpopulation* effects associated with the X chromosome (i.e., survivorship differences between IS and SI males [Fig. 1], or between flies carrying X chromosomes from I and S regimes [Fig. 3]). In the Hybrid Experiment, in each of the four blocks, for every cross, we set up three replicate cages each containing 50 infected males and 50 infected females. In the X‐Cloning Experiment, we sampled 80 X chromosomes from the I regime and 80 X chromosomes from the S regime. Corresponding to each X‐line, we assayed the survivorship of 24 individuals per sex. As a result, our findings provide strong evidence that X chromosomes had very little, if any, contribution to the *evolved differences* in postinfection survivorship between I and S regimes, despite strong directional selection in the I populations. This implies that the ancestral BRB populations, and by extension I_1‐4_ and S_1‐4_ populations, had inadequate X‐linked genetic variation. Further, the lack of X‐linked effects in females could also be an artifact of genes on the sampled X chromosomes always being expressed in a heterozygous state in females in the X‐Cloning Experiment, making large recessive effects invisible. However, our finding that immunocompetence‐related genes selected in the I populations are, on average, partially dominant in females makes this scenario improbable. Nevertheless, to rule out the effect of uncontrolled autosomal variation and recessive variation in females, a confirmatory test merging our cloning approach with that of Hill‐Burns and Clark (2009) would be required, where target X chromosomes are expressed both in homozygous and heterozygous states, in multiple isogenic autosomal backgrounds drawn from the ancestral populations.

Our results are consistent with recent studies using laboratory populations of *D. melanogaster *that did not find unequivocal evidence for enrichment of X‐linked SA polymorphisms (Abbott et al. 2020; Lund‐Hansen et al. 2020). Ruzicka and Connallon (2020) showed that the signal of sexual antagonism is stronger on X chromosomes than autosomes, even when both are equally permissive for SA polymorphisms. They argued that studies that do find an overrepresentation of SA variation on the X chromosome (Gibson et al., 2002; Pischedda and Chippindale 2006; Foerster et al. 2007; Fedorka and Mousseau 2004; Calsbeek and Sinervo 2004; Long et al. 2012; Ruzicka et al. 2019; Lucotte et al. 2016) are consistent with X chromosomes and autosomes being equally permissive for maintenance of SA polymorphisms. Rice (1984), while arguing that X chromosomes are hotspots of SA variation, assumed that female‐beneficial alleles are at least partially dominant, and dominance coefficients are identical between the sexes. Studies that relaxed these assumptions by assuming additivity (Pamilo 1979), by exploring a wide range of dominance conditions (Curtsinger,1980; Patten and Haig 2009), or by investigating SSDRs (Fry 2010; Connallon and Clark 2012) showed that X chromosomes need not always be enriched for SA polymorphisms. In light of our results indicating sex‐specific dominance for surviving infection, absence of an effect of the X chromosome is quite unsurprising. Connallon and Clark (2012) also argued that the proportion of SA polymorphisms that are X‐linked will depend on a host of variables including sex‐biased mutation rates and the effective population size of the X chromosome relative to the autosomes. We hypothesize that in the wild ancestors of our experimental populations, due to sex‐specific dominance and/or other factors, immunocompetence‐related genetic variation was maintained on the autosomes, but depleted from the X chromosome.

Although both experiments are consistent in their findings, the X‐Cloning Experiment was performed around 90 generations after the Hybrid Experiment. However, this is unlikely to affect our broad conclusions. In the intervening ∼90 generations, I populations consistently exhibited higher survivorships than S populations post infection by *P. entomophila* (Ravikumar 2016; Shit 2019). Additionally, both experiments agree in their findings regarding the absence of contribution of the X chromosome in the evolution of the I populations. Had there been a small effect of the X‐chromosome at the time of the Hybrid Experiment which went undetected, it should have been amplified in the subsequent ∼90 generations of strong directional selection. However, no such effect was detected in the X‐Cloning Experiment. 

Another caveat of this study is that our results are based only on survivorship post infection by *P. entomophila*. Although this may not necessarily reflect all the different components of immunocompetence, survivorship post infection by *P. entomophila*, a bacterium originally isolated from a wild *D. melanogaster* female (Dieppois et al. 2015), is an ecologically relevant measure of immunocompetence in our system. Surviving infection is a crucial determinant of an organism's fitness in an environment where infection is guaranteed, such as the I populations. Additionally, in *D. melanogaster*, postinfection survivorship is strongly correlated with other measures of immunocompetence such as antimicrobial peptides (Schwenke and Lazzaro 2017). Nevertheless, we urge caution in extrapolating our findings to immunity in general.

## Conclusion

Our study highlights two important aspects of the evolutionary genetics of immunocompetence in *D. melanogaster*. First, we show that the X chromosome may not play an important role in aiding populations to adapt to pathogenic infection. Second, we report evidence of sex‐specific dominance in *D. melanogaster *with respect to surviving pathogenic infection by *P. entomophila*. We also highlight sex‐specific dominance as a potential mechanism generating sex differences in immunocompetence.

## AUTHOR CONTRIBUTIONS

VG standardized and set up the I and S selection regimes, carried out the principal experimental evolution work, and highlighted the potential role of the X chromosome. NGP, ZAS, SV, and MGA designed the Hybrid Experiment. MGA, ZAS, and SV executed the Hybrid Experiment. AA, J, and NGP designed the X‐Cloning Experiment. AA, J, MGA, MK, and TSC carried out the X‐Cloning Experiment. MGA, AA, and NGP analyzed the data. All authors contributed to interpreting the results. MGA and AA wrote the first draft of the manuscript with critical inputs from SZA, J, and NGP. All authors reviewed the manuscript.

## Conflict of Interest

The authors declare no conflict of interest.

## DATA ARCHIVING

The Dryad Digital Repository link for the data used in this study is as follows: https://doi.org/10.5061/dryad.0cfxpnw30.

## Supporting information


**Figure S1 **A schematic for the crossing scheme used in the X‐Cloning Experiment.
**Figure S2A** Effect of cross and sex on proportion survivorship at the end of the observation window in the Hybrid Experiment plotted separately for each block.
**Figure S2B** Effect of cross and sex on proportion survivorship at the end of the observation window in the Hybrid Experiment when data from all four blocks were plotted together.
**Figure S3** Effect of cross (II, red; IS, brown; SI, purple; SS, blue) and sex (solid, females; dashed, males) on survivorship post infection in the Hybrid Experiment, when data from all four blocks are combined.
**Figure S4**. Point estimates and 95% confidence intervals (CIs) for the dominance coefficient for proportion survivorship in females (Df), the dominance coefficient for proportion survivorship in males (D_m_) and the difference in the dominance coefficients for proportion survivorship in females and males (i.e., *D*_f_ – D_m_) for each of the four blocks.
**Figure S5A **Effect of selection regime and sex on proportion survivorship at the end of the observation window for the X‐Cloning Experiment when plotted separately for each block.
**Figure S5B **Effect of selection regime and sex on proportion survivorship at the end of the observation window for the X‐Cloning Experiment when data from all four blocks were plotted together.
**Figure S6**. Effect of selection regime (red, I; blue, S) and sex (solid, females; dashed, males) on survivorship post infection in the X‐Cloning Experiment when data from all four blocks were plotted together.
**Figure S7A **Effect of selection regime and sex on median time to death in the X‐Cloning Experiment when plotted separately for each block.
**Figure S7B **Effect of selection regime and sex on median time to death in the X‐Cloning Experiment when data from all four blocks were plotted together.
**Figure S8. **Posterior distributions of X‐linked heritabilities for standardized median time to death post infection for I females (A), I males (B), S females (C), and S males (D), and posterior distributions for the difference in male and female heritabilities for standardized median time to death for the I regime (E) and the S regime (F), obtained from the MCMCglmm models for the X‐Cloning Experiment.
**Figure S9A**. Interaction plot for median time to death post infection for various X‐lines in males and females. Points connected by lines belong to the same X‐line.
**Figure S9B**. Interaction plot for proportion survivorship 96 hours post infection for various X‐lines in males and females. Points connected by lines belong to the same X‐line.
**Figure S10. **Correlation between median time to death of males and females from the same X lines in (A) I1, (B) S1, (C) I2, (D) S2, (E) I3, (F) S3, (G) I4, and (H) S4.
**Figure S11. **Correlation between proportion survivorship of males and females from the same X lines in (A) I1, (B) S1, (C) I2, (D) S2, (E) I3, (F) S3, (G) I4, and (H) S4.
**Table S1 **Tukey's HSD for proportion survivorship in the Hybrid Experiment.
**Table S2** Summary of the Hybrid Experiment results (A) Estimates and 95 % confidence intervals (CIs) for relative hazard rates (i.e., the exponent of the coefficients) corresponding to various fixed parameters of the Cox's proportional hazards model. Hazard rates are expressed relative to the default level of that fixed factor.
**Table S3** Summary of the X‐Cloning Experiment results (A) Estimates and 95 % confidence intervals (CIs) for relative hazard rates (i.e., the exponent of the coefficients) corresponding to various fixed parameters of the Cox's proportional hazards model.
**Table S4 **Deviance Information Criteria (DIC) for the MCMCglmm models for the X‐Cloning Experiment to test for the effect of X‐line.
**Table S5A. **Results of correlation analysis between median time to death of males and females of the same X line by (i) Linear Model and (ii) Spearman's rank correlation.
**Table S5B. **Results of correlation analysis between proportion survivorship of males and females of the same X line by (i) Linear Model and (ii) Spearman's rank correlation.
