# Supplementary material for: Experimental evolution reveals sex‐specific dominance for surviving bacterial infection in laboratory populations of Drosophila melanogaster
Source: Evol Lett. 2021 Oct 14;5(6):657–71. doi: 10.1002/evl3.259 (PMC8645198; doi:10.1002/evl3.259)
Supplement: Supplementary file 1 — Figure S1 A schematic for the crossing scheme used in the X‐Cloning Experiment. Figure S2A Effect of cross and sex on proportion survivorship at the end of the observation window in the Hybrid Experiment plotted separately for each block. Figure S2B Effect of cross and sex on proportion survivorship at the end of the observation window in the Hybrid Experiment when data from all four blocks were plotted together. Figure S3 Effect of cross (II, red; IS, brown; SI, purple; SS, blue) and sex (solid, females; dashed, males) on survivorship post infection in the Hybrid Experiment, when data from all four blocks are combined. Figure S4. Point estimates and 95% confidence intervals (CIs) for the dominance coefficient for proportion survivorship in females (Df), the dominance coefficient for proportion survivorship in males (Dm) and the difference in the dominance coefficients for proportion survivorship in females and males (i.e., D f – Dm) for each of the four blocks. Figure S5A Effect of selection regime and sex on proportion survivorship at the end of the observation window for the X‐Cloning Experiment when plotted separately for each block. Figure S5B Effect of selection regime and sex on proportion survivorship at the end of the observation window for the X‐Cloning Experiment when data from all four blocks were plotted together. Figure S6. Effect of selection regime (red, I; blue, S) and sex (solid, females; dashed, males) on survivorship post infection in the X‐Cloning Experiment when data from all four blocks were plotted together. Figure S7A Effect of selection regime and sex on median time to death in the X‐Cloning Experiment when plotted separately for each block. Figure S7B Effect of selection regime and sex on median time to death in the X‐Cloning Experiment when data from all four blocks were plotted together. Figure S8. Posterior distributions of X‐linked heritabilities for standardized median time to death post infection for I females (A), I males (B), S fem [file EVL3-5-657-s001.docx]

**Supplementary Information**

**A) Details of Fly Populations and Experimental Protocols**

**Fly populations:** All fly populations detailed below are maintained on standard banana-yeast-jaggery food unless specified otherwise.

Ancestral populations: Blue Ridge Baselines (BRB1-5) are large, independent, outbred populations of *D. melanogaster* that trace their ancestry to 19 iso-female lines, each of which was set up in 2010 by collecting a single wild, inseminated female from the Blue Ridge Mountains, USA. These lines were gifted to us by Dr. Daniel Promislow in 2010. After 6 generations in our laboratory, we established a single large population, called the Blue Ridge Baseline (BRB), by mixing together 100 males and 100 females from each of these 19 lines. This population was maintained under standard laboratory maintenance conditions (described below for BRB1-5 populations) for 10 generations, after which it was split into five replicate populations BRB1-5. The population maintenance for BRB1-5 populations has been described in detail by Singh et al. (2015).  To summarise, the five BRB populations are maintained on a 14-day discrete generation cycle, 12:12 Light:Dark regime, on banana-jaggery-yeast food, 25°C and 60-70% Relative Humidity. For each BRB population eggs are collected at a density of 70 eggs per vial containing 8-10 ml banana-jaggery-yeast food. Forty such vials are set up for every BRB population, yielding a total population size of around 2800 eggs. On the 12^th^ day post egg collection, by which time all eggs develop into adults, flies from all forty vials of a population are transferred to a Plexiglas cage (24×19×14 cm). These flies are supplied with a fresh food plate smeared with an ad-libitum supplement of live yeast in the form of a paste. On the 14^th^ day post egg collection, each BRB population cage is given a fresh food plate for oviposition. Eggs laid on these plates over the next 18 hours are used to start the next generation. Populations used in this study were derived from only four BRB (BRB_1-4_) populations.

Selection Regimes: We used four replicate populations (I_1-4_) selected against *Pseudomonas entomophila* strain L48 and their respective controls (S_1-4_) which have been described in detail elsewhere (Gupta 2016; Gupta et al. 2016). These populations were derived from the BRB_1-4_ populations 22 generations after the five BRB populations were established by splitting the single BRB population (see above). Each BRB population was used to derive one I population and one S population. To summarise the selection regimes, for each of the I populations, 2-3 day old adults (150 males and 150 females) are infected (see below for protocol) with *P. entomophila.* For each of the S populations, 100 males and 100 females are subjected to a “sham-infection” treatment (see below). Post infection or sham-infection flies from each of the I and S populations are transferred to Plexiglas cages (14 cm × 16 cm × 13 cm) and are provided with food plates. These food plates are replaced with fresh ones after two days. In I populations, around 33% flies die over 96 hours after infection. The S populations experience no mortality in the same window of 96 hours. Eggs laid in the 18 hour window starting at 96 hours post infection/sham-infection, contribute to the next generation. For each of the I and S populations, 10 vials (90 mm length × 2.5 mm diameter), each containing 70 eggs in 8-10 ml banana-jaggery-yeast food, are set up every generation. It is important to note that there is strong selection in the I populations to survive the infection for at least 96 hours. Therefore, in our survivorship assays (see below), we record mortality for 96 hours post infection so that our assays reflect a measure of survivorship that is relevant to our selection regimes. Furthermore, mortality due to bacterial infection also ceases after 96 hours post-infection. Also note that the I populations start with more flies than the S populations so that after pathogen-induced mortality, both I and S populations have comparable number of flies alive, ie around 200 adults per population. Populations with a common subscript are always handled on the same day and are related by ancestry (I_1-4_ and S_1-4_ populations were derived from the respective BRB_1-4_ population). They are, therefore, treated as statistical “blocks”. Thus, the entire evolution experiment is replicated four times over. The I populations have been previously shown to have evolved superior immunocompetence in terms of survivorship post infection relative to the S populations (Gupta et al. 2016).

Clone Generators (CG): Clone generator females carry a compound X [C(1)DX y f] chromosome, Y chromosome, and a homozygous viable translocation of the two autosomes [T(2;3) rdgC st in ripP bwD]. Males have an X [sn su(b)] chromosome, Y chromosome and the same translocated autosomes. In this system, females inherit the compound X chromosome from their mother and a Y chromosome from their father. Males inherit the Y chromosome from their mother and the X chromosome from their father. The system has been described in detail by Rice (1996). Clone generators are maintained on standard cornmeal-yeast-molasses food.

DxBRB: The DxBRB population was created by introgressing the compound X chromosome into BRB1. This population is maintained like BRB populations.

**Protocol for infections/sham infections**: An overnight culture of *P. entomophila* strain L48 was set up by inoculating the frozen glycerol stock of the bacterium into 10 ml Luria Bertani (LB) Broth (HiMedia). From this culture, a secondary culture was set up the next morning, and allowed to grow for 3-4 hours. Subsequently, the secondary culture was centrifuged and the bacterial pellet was suspended in appropriate volume (depending on the required OD_600_) of 10 mM sterile MgSO_4_. The infection protocol involved pricking CO_2_anaesthetised flies with a needle (Minutein pin 0.1 mm, Fine Science Tools, CA) dipped in the bacterial suspension). For sham infections, the pricking protocol was similar, except we dipped the needle in a sterile 10 mM MgSO_4_ solution.

**Protocol for the Hybrid Experiment:**

This experiment was carried out between 65 and 75 generations of selection. For each block, we first collected eggs from I and S flies subjected to one generation of common rearing, which involved transferring them to Plexiglas cages (14 cm × 16 cm × 13 cm) without any infection (to remove non-genetic parental effects). Ten vials of 70 eggs each were set up per population. Adult males and females that emerged from these vials were collected as virgins in sex-separated vials (10 individuals per vial) and were combined in Plexiglas cages to set up the following crosses of 100 pairs each.

1. I ♀ × I ♂ (II)
2. S ♀ × S ♂ (SS)
3. I ♀ × S ♂ (IS)
4. S ♀ × I ♂ (SI)

In order to generate the F1 progeny to be used in our experiments, we collected 10 vials each containing 70 eggs in 8-10 ml food for every cross. On the 12^th^-day post egg collection, for every cross, we set up three replicate cages (14 cm × 16 cm × 13 cm), each containing 50 males and 50 females infected with *P. entomophila*(OD_600_=1.5). Infections for each of the three replicates for each cross were handled by a different infector, allowing us to incorporate a random effect corresponding to “infector” in our analyses (see below). Additionally, for each cross, we also set up a cage containing 50 male and 50 females that were sham-infected. We monitored mortality over the next 96 hours. Food plates in the cages were replaced with fresh ones two days after the cages were set up.

**Protocol for the X-cloning Experiment:**

Cloning the X chromosomes:

We sampled 20 random X chromosomes from each of the I and S populations. In order to express the X chromosome from I and S populations in a neutral common chromosomal background, the following crosses were set up over 4 generations (See Figure S1 for a detailed schematic):

1. I/S♂ × CG♀ 🡪 Progeny1_(Males)_ + other progeny
2. Progeny1♂ ×  DxBRB♀ 🡪 Progeny2_(Brown-eyed Males)_+ other progeny
3. Progeny2♂ ×  DxBRB♀ 🡪 Progeny3_(Red-eyed Males)_+ other progeny
4. Progeny3♂ ×  DxBRB♀ 🡪 Experimental flies_(Males)_+ other progeny 
   Progeny3♂ × BRB_1_♀ 🡪 Experimental flies_(Females)_+ other progeny

20 Males from each I and S population were crossed to 10 Clone Generator females each. Three males resulting from each of these crosses were crossed to 5 virgin DxBRB females. Three brown-eyed males resulting from each of these crosses were again crossed to 5 virgin DxBRB females. In order to generate male and female flies to be used in the survivorship assay, 3 red-eyed males resulting from the each of the previous crosses were crossed to 5 virgin DxBRB and 5 BRB females respectively. The experimental flies carry an X chromosome from I or S populations, but the rest of the genome is from BRB (other than the unmanipulated fourth chromosome). Since our crossing scheme involved introgression of the BRB autosomal background three times, a majority of fourth chromosomes in our X-lines were also expected to be from the ancestral BRB population. Note that these females carried one target X chromosome (from I or S populations) and one X chromosome from BRB_1_. A single vial per X-line was maintained for all crosses. Egg densities were maintained such that there were 70 viable eggs per vial. Crosses were set on the 12^th^day post egg collection.

Survivorship Assay:   On the 11^th^day post egg-collection (of the 4^th^ cross), flies were sorted into same sex vials (10 per vial). On the 12^th^ day, flies were infected with *P. entomophila* (OD_600_=1) and transferred to fresh vials. 3 vials per X-line (8 flies per vial) per sex were set up for the infected treatment, each handled by a different infector. We also set up one vial per X-line of sham controls. Mortality was monitored over a 96-hour period. The surviving flies were transferred to fresh food vials after 2 days.

**B) Details of Statistical Analyses**

In all analyses, data from the sham-infection treatment were excluded, as in both of our experiments there was nomortality due to sham-infection. All analyses were performed in R (Version 4.0.2).

A) Hybrid Experiment:

We performed three different analyses for this experiment.

First, for each combination of cross, sex, infector and block we calculated the proportion of flies alive at the end of the 96-hour observation window. Using this proportion as the unit of analysis we fit the following linear mixed effects model using the R packages “lme4” and “lmerTest”:

Proportion Survivorship ~ Cross + Sex + Cross:Sex + (1 | Block) + (1 | Sex:Block) + (1 | Cross:Block) + (1|Infector).

This model yielded a singular fit and could not estimate the variance associated the term (1|Cross:Block). Removing this term did not affect in any way the estimates corresponding to the rest of the terms. Therefore, we continue to retain the full model.

Post-hoc comparisons using Tukey’s HSD were implemented with the R package “emmeans” (Lenth 2020).

Second, we used the R package “coxme” and fit the following Cox’s proportional hazards model:

Time to death ~ Cross + Sex + Cross:Sex +  (1 | Block/Sex/Cross) + (1 | Infector)

Third, we used the R package “lme4” to fit the following model using logistic regression on the status (dead or alive) of each individual fly at the end of the 96-hour observation window.

Status ~ Cross + Sex + Cross:Sex  + (1 | Block) + (1|Block:Cross) + (1 | Infector)

None of our analyses could detect a difference between the survivorship post infection in the two reciprocal hybrid crosses (see Results), indicating an absence of contribution by the X-chromosome. However, our results (Table 1A, Table S1, Figure 1, Figure S2) suggested that differences in the two sexes were primarily driven by differences between the two sexes within the hybrid crosses (IS and SI) but not within the parental crosses (II and SS). Therefore, in order to investigate the possibility of sex-specific dominance, we also calculated an estimate for the dominance coefficient for proportion survivorship for both sexes. Since neither of our analyses could distinguish between IS and SI (see results), we used the average proportion survivorship of IS and SI of the ‘n’th block as the average heterozygote proportion survivorship for that block. We used the following expression to calculate the dominance coefficient:

D = (0.5×(P_IS_+P_SI_)−P_SS_)/(P_II_−P_SS_), where P_II_, P_SS_, P_IS_ and P_SI_ represent proportion survivorships in the crosses II, SS, IS and SI respectively. (Ewens, 2004, adapted from fitness scheme 1.25b, Section 1.4). To test for departure from additivity and sex-specific dominance, we used a stratified bootstrap approach using the R package “boot” (Canty and Ripley, 2020). We sampled with replacement within each combination of Block, Sex, Cross and Infector to generate 10000 data-sets. For each data set, we measured the dominance coefficient for proportion survivorship in females (Df), the dominance coefficient for proportion survivorship in males (Dm) as well as the difference in the two (Df-Dm), separately for each of the four blocks, as well as for the entire data. We then calculated 95% confidence intervals (CIs) for Df, Dm and Df-Dm, separately for each block, as well as for the entire data. To test for departure from additivity, we examined if the CIs for Dm and Df overlapped with 0.5. To test for sex-specific dominance, we examined if the CIs for Df-Dm overlapped with 0.

B) X-cloning Experiment:

We performed four different analyses for this data.

We calculated proportion survivorship at the end of the 96-hour observation window for each combination of block, selection regime, sex and X chromosome line. For this purpose, we pooled the data from the three vials for each X chromosome line.  We then fit the following linear mixed-effects model using the R package lme4:

Y ~ SelectionRegime + Sex + SelectionRegime:Sex  +  (1 | Block) +  (1 | X-line:SelectionRegime:Block)

We calculated the median time to death for each vial and fit the following linear mixed-effects model using lme4:

Y ~ SelectionRegime + Sex + SelectionRegime:Sex  + (1 | Infector) + (1 | Block) +    (1 | X-line:SelectionRegime:Block)

We fit the following logistic regression on the status (dead or alive) of each fly at the end of the 96-hour observation window:

Status ~ Selection Regime + Sex + SelectionRegime:Sex + (1 | Infector) + (1 | Block) + (1 | X-line:Block:SelectionRegime)

We fit the following Cox proportional hazards model:

Time to death ~ SelectionRegime+ Sex + SelectionRegime:Sex +(1 | Block/SelectionRegime/Xline) + (1 | Infector)

Additionally, we used a Bayesian approach to investigate X-linked genetic variation associated with surviving infection by *P. entomophila*in I and S selection regimes. To that end we used the R package “MCMCglmm” that incorporates Markov Chain Monte Carlo sampling techniques to fit the following model separately for I and S selection regimes: T_ijkm_ ~ S_i_ + L_ij_ + B_ik_ + ε_ijkm_where T_ijkm_ is the scaled and centred median time to death of vial m of sex i, X-line j and block k. The fixed effect of sex is represented by S_i_. L_ij_ corresponds to the random effect of X-line. It is modeled to follow a bivariate normal distribution with a mean 0 and a variance-covariance matrix given by X-linked additive genetic variance in standardised median time to death for females (V_x-f_), X-linked additive genetic variance in standardised median time to death for males (V_x-m_) and the intersexual X-linked additive genetic covariance (Cov_x-mf_) for standardised median time to death. B_ik_ represents random blocks and is modeled to follow a normal distribution with a mean 0 and variances given by sex-specific variances in standardised median time to death corresponding to block. ε_ijkm_ represents sex-specific residuals which too are modeled to follow a normal distribution with mean 0 and variances given by residual variances in standardised median time to death for males (V_res-m_) and females (V_res-f_). Using these estimates, we calculated the following quantitative genetic parameters for median time to death: 1. X-lined male heritability given by (V_x-m_)/((V_x-m_) + (V_res-m_)), 2. X-linked female heritability given by 2×(V_x-f_)/((V_x-f_) + (V_res-f_)) (Griffin et al., 2016) and 3. X-linked intersexual genetic correlation given by (Cov_x-mf_)/(sqrt(V_x-m_) × sqrt(V_x-f_)). Using the posterior MCMC distributions, we calculated 95% credible intervals for male heritability, female heritability, the difference between male and female heritabilities and the intersexual genetic correlation for standardized median time to death separately for each selection regime. To explicitly test for the effect of X-line, for both I and S selection regimes, we also fit a separate null model that lacked the term L_ij_. For each selection regime, we compared the Deviance Information Criterion (DIC) between the model that incorporated L_ij_ with that of the null model. For each model, the simulation was run for 100000 iterations, with a burn-in period of 25000 iterations and sampling interval set to 50 iterations.

We calculated the average median time to death and proportion survivorship for each X-line in both the sexes. Average median time to death was calculated as the mean of median time to death across the all vials of an X-line.

For these two read-outs of survivorship, we calculated the correlation between males and females using two methods

First, we fit the following linear model separately for each combination of selection regime and block:

FemaleMeasure ~ MaleMeasure

Second, we also calculated correlation using Spearman’s Rank Correlation.

**C) Supplementary Figures and Tables**

**Figure S1**A schematic for the crossing scheme used in the X-Cloning Experiment. The translocated 2nd and 3rd chromosomes are represented by a black bar since they must be inherited together. X, Y, II and III represent the respective chromosomes. X.X represents the compound X chromosome. The subscript represents the fly stock/selection regime from which the chromosome originates.

**Figure S2A** Effect of cross and sex on proportion survivorship at the end of the observation window in the Hybrid Experiment plotted separately for each block. Red boxes represent females while blue boxes indicate males.

 

**Figure S2B** Effect of cross and sex on proportion survivorship at the end of the observation window in the Hybrid Experiment when data from all four blocks were plotted together. Red boxes represent females while blue boxes indicate males.


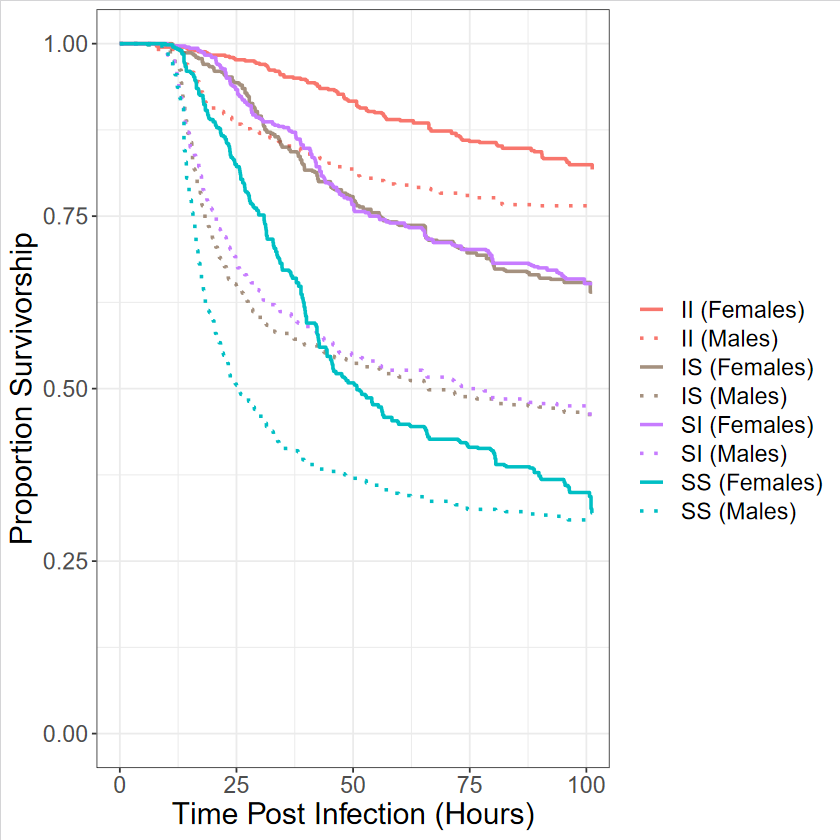


**Figure S3** Effect of cross (II-red, IS-brown, SI-purple, SS-blue) and sex (solid-females, dashed-males) on survivorship post infection in the Hybrid Experiment, when data from all four blocks are combined. The curves show survival of the F1 progeny as a function of time. The first letter indicates the maternal selection regime and the second, the paternal.

**Figure S4.** Point estimates and 95% confidence intervals (CIs) for the dominance coefficient for proportion survivorship in females (Df), the dominance coefficient for proportion survivorship in males (Dm) and the difference in the dominance coefficients for proportion survivorship in females and males (ie, Df-Dm) for each of the four blocks. Confidence intervals were generated by bootstrapping the data-set 1000 times (see the section on Statistical Analysis). Note that the CIs for Df and Dm do not overlap with 0.5 and the CIs for Df-Dm do not overlap with 0.

**Figure S5A**Effect of selection regime and sex on proportion survivorship at the end of the observation window for the X-cloning Experiment when plotted separately for each block. Red boxes represent females while blue boxes indicate males.


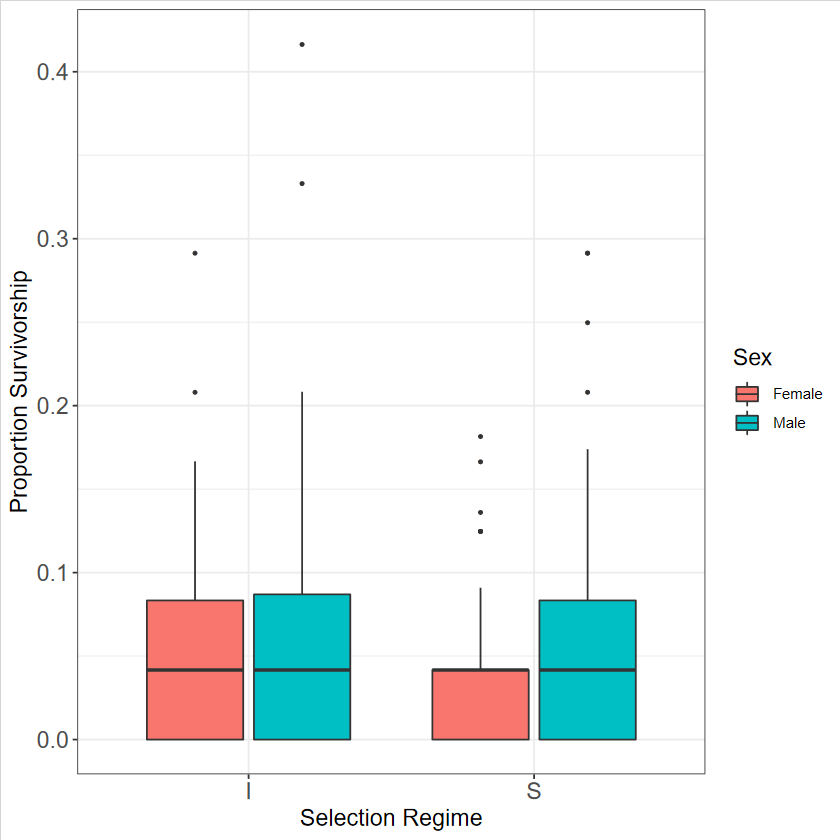


**Figure S5B**Effect of selection regime and sex on proportion survivorship at the end of the observation window for the X-cloning Experiment when data from all four blocks were plotted together. Red boxes represent females while blue boxes indicate males.


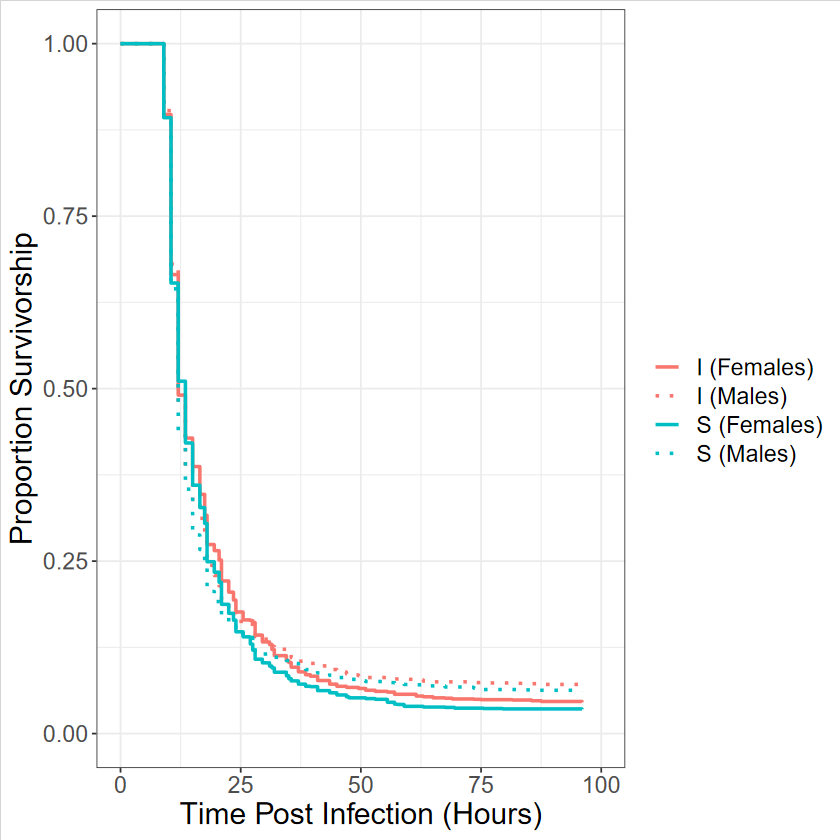


**Figure S6.** Effect of selection regime (red-I, blue-S) and sex (solid-females, dashed-males) on survivorship post-infection in the X-Cloning Experiment when data from all four blocks were plotted together. I and S flies carry the X chromosome from the respective selection regime but share the rest of the genome, which comes from a baseline population.

**Figure S7A**Effect of selection regime and sex on median time to death in the X-cloning Experiment when plotted separately for each block. Red boxes represent females while blue boxes indicate males.


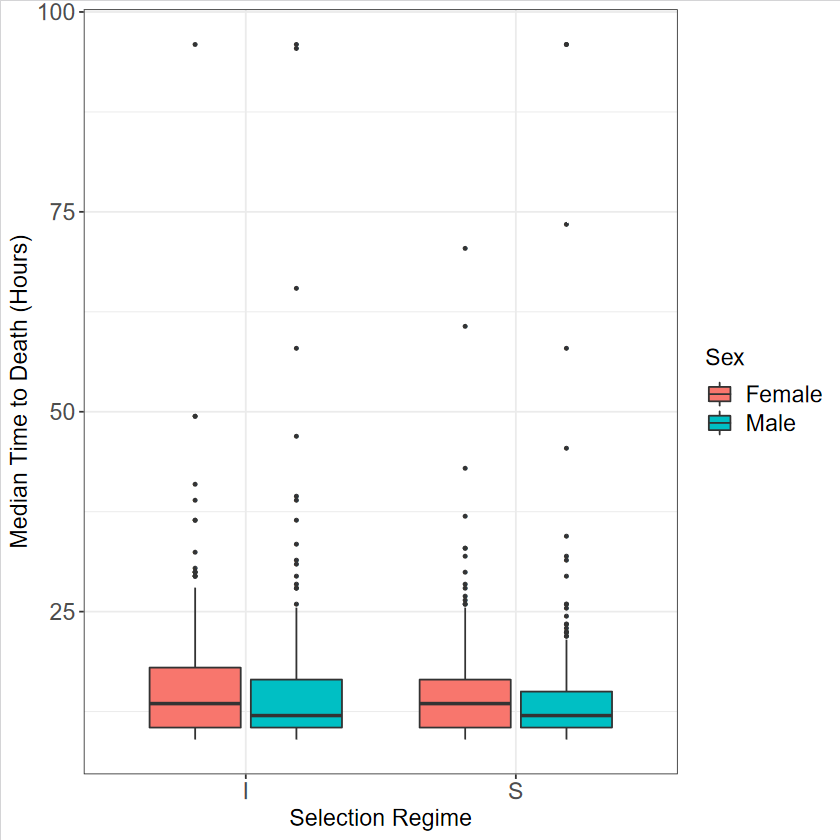


**Figure S7B**Effect of selection regime and sex on median time to death in the X-cloning Experiment when data from all four blocks were plotted together. Red boxes represent females while blue boxes indicate males.


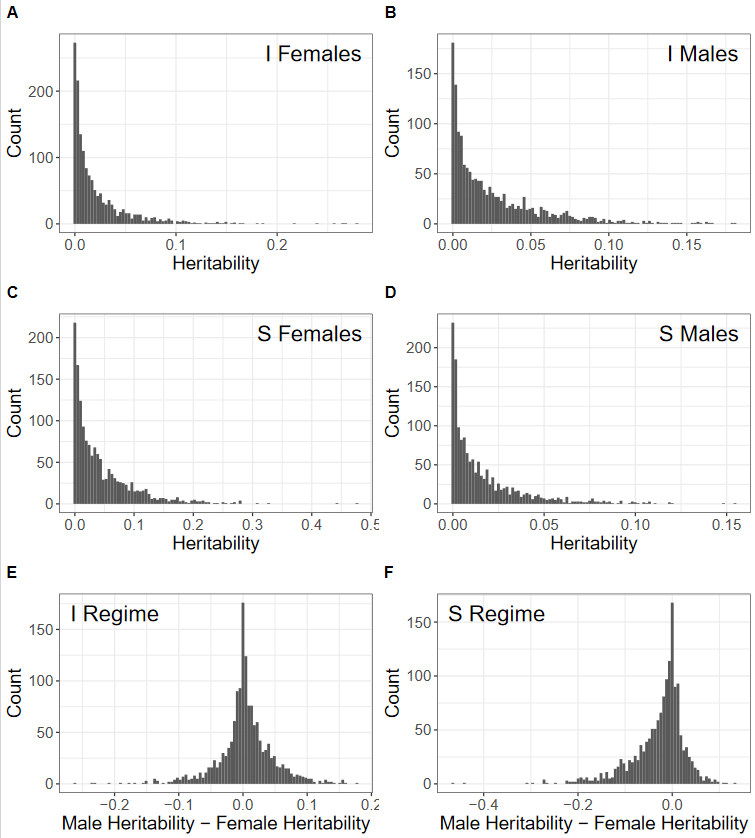


**Figure S8.**Posterior distributions of X-linked heritabilities for standardised median time to death post infection for I females (A), I males (B), S females (C) and S males (D), and posterior distributions for the difference in male and female heritabilities for standardized median time to death for the I regime (E) and the S regime (F), obtained from the MCMCglmm models for the X-cloning Experiment. Each distribution was plotted using 1500 data points.

**Figure S9A.** Interaction plot for median time to death post infection for various X-lines in males and females. Points connected by lines belong to the same X-line. Each panel represents data for X-lines from one of the 8 experimental populations. The four panels on the left represent four I populations (I_1-4_) and the panels on the right represent four S populations (S_1-4_).

**Figure S9B.** Interaction plot for proportion survivorship 96 hours post infection for various X-lines in males and females. Points connected by lines belong to the same X-line. Each panel represents data for X-lines from one of the 8 experimental populations. The four panels on the left represent four I populations (I_1-4_) and the panels on the right represent four S populations (S_1-4_). (Note: There appear to be fewer than 20 points in many populations, as many X-lines had identical proportion survivorships, resulting in overlapping points.)


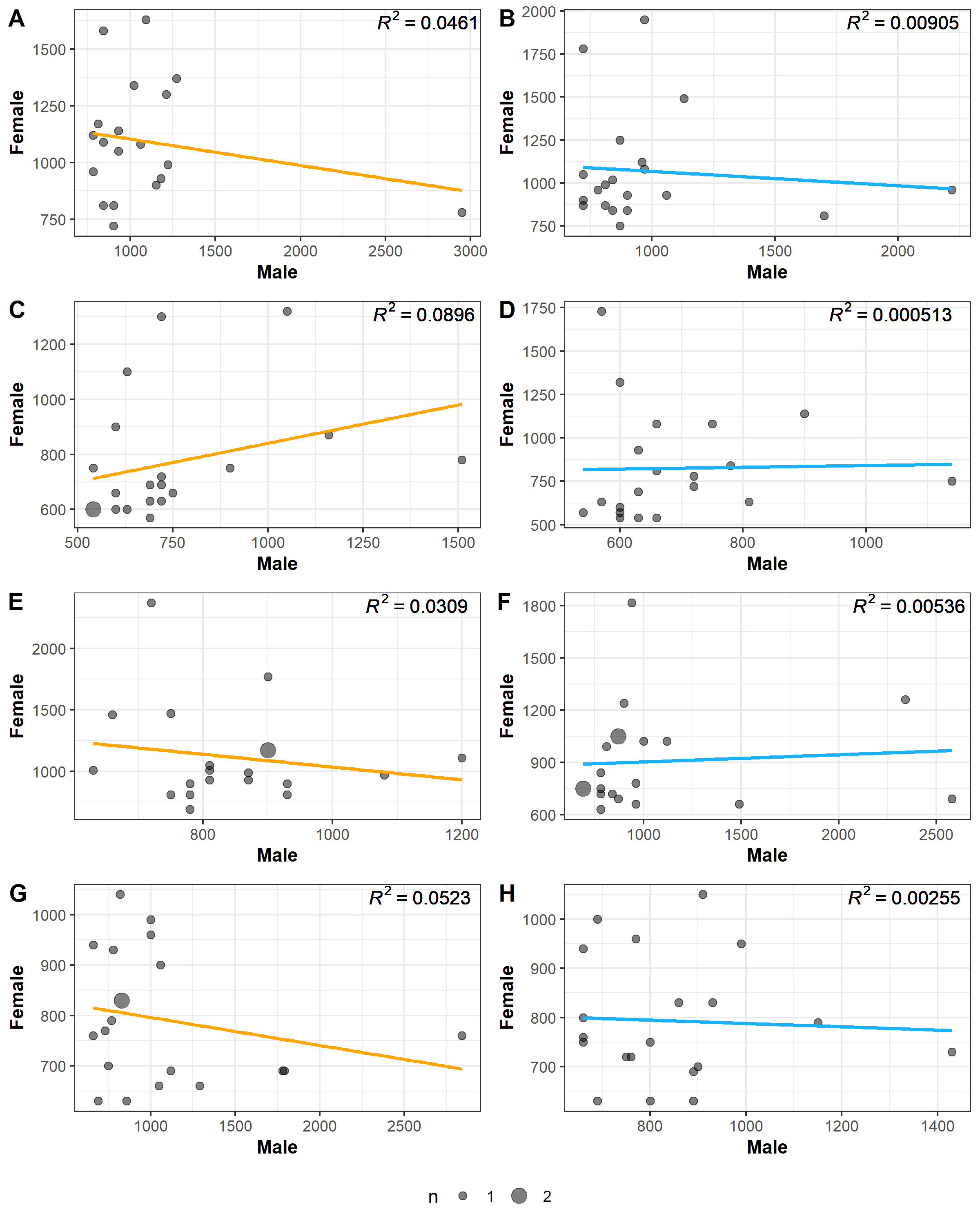


**Figure S10.**Correlation between median time to death of males and females from the same X lines in A) I1, B) S1, C) I2, D) S2, E) I3, F) S3, G) I4, H) S4

 
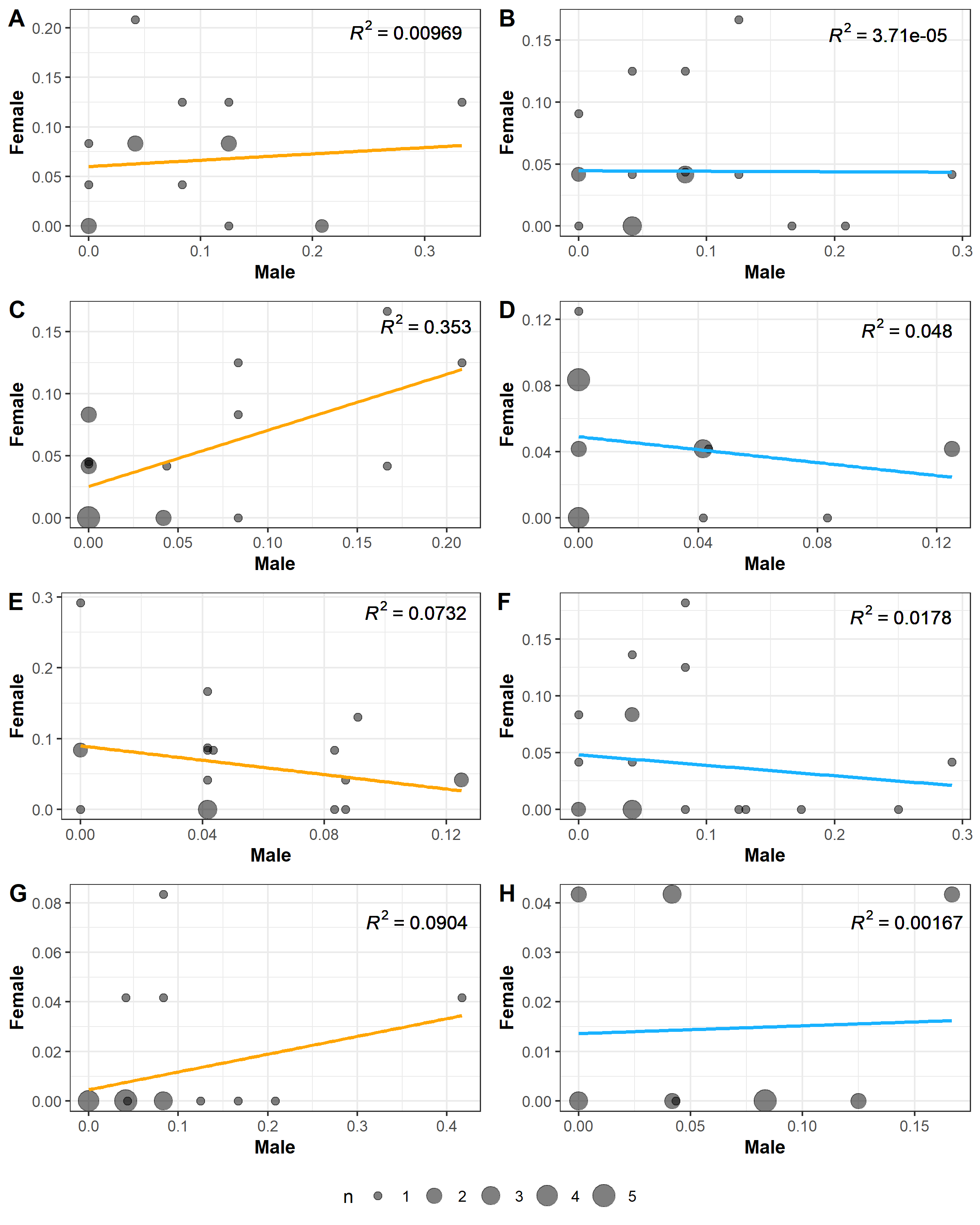


**Figure S11.**Correlation between proportion survivorship of males and females from the same X lines in A) I1, B) S1, C) I2, D) S2, E) I3, F) S3, G) I4, H) S4

**Table S1**Tukey’s HSD for proportion survivorship in the Hybrid Experiment

| contrast | | estimate | SE | df | t.ratio | p value |
| --- | --- | --- | --- | --- | --- | --- |
| II,Female | IS,Female | 0.1750 | 0.0463 | 31.9 | 3.783 | **0.0132** |
| II,Female | SI,Female | 0.1683 | 0.0463 | 31.9 | 3.639 | **0.019** |
| II,Female | SS,Female | 0.4800 | 0.0463 | 31.9 | 10.376 | **<.0001** |
| II,Female | II,Male | 0.0617 | 0.0505 | 19.5 | 1.222 | 0.9157 |
| II,Female | IS,Male | 0.3617 | 0.0505 | 15.2 | 7.165 | **0.0001** |
| II,Female | SI,Male | 0.3550 | 0.0505 | 15.2 | 7.033 | **0.0001** |
| II,Female | SS,Male | 0.5150 | 0.0505 | 15.2 | 10.202 | **<.0001** |
| IS,Female | SI,Female | -0.0067 | 0.0463 | 31.9 | -0.144 | >0.9999 |
| IS,Female | SS,Female | 0.3050 | 0.0463 | 31.9 | 6.593 | **<.0001** |
| IS,Female | II,Male | -0.1133 | 0.0505 | 15.2 | -2.245 | 0.3795 |
| IS,Female | IS,Male | 0.1867 | 0.0505 | 19.5 | 3.698 | **0.026** |
| IS,Female | SI,Male | 0.1800 | 0.0505 | 15.2 | 3.566 | **0.0433** |
| IS,Female | SS,Male | 0.3400 | 0.0505 | 15.2 | 6.735 | **0.0001** |
| SI,Female | SS,Female | 0.3117 | 0.0463 | 31.9 | 6.737 | **<.0001** |
| SI,Female | II,Male | -0.1067 | 0.0505 | 15.2 | -2.113 | 0.4486 |
| SI,Female | IS,Male | 0.1933 | 0.0505 | 15.2 | 3.83 | 0.0265 |
| SI,Female | SI,Male | 0.1867 | 0.0505 | 19.5 | 3.698 | **0.026** |
| SI,Female | SS,Male | 0.3467 | 0.0505 | 15.2 | 6.867 | **0.0001** |
| SS,Female | II,Male | -0.4183 | 0.0505 | 15.2 | -8.287 | **<.0001** |
| SS,Female | IS,Male | -0.1183 | 0.0505 | 15.2 | -2.344 | **0.3319** |
| SS,Female | SI,Male | -0.1250 | 0.0505 | 15.2 | -2.476 | 0.2748 |
| SS,Female | SS,Male | 0.0350 | 0.0505 | 19.5 | 0.693 | 0.9962 |
| II,Male | IS,Male | 0.3000 | 0.0463 | 31.9 | 6.485 | **<.0001** |
| II,Male | SI,Male | 0.2933 | 0.0463 | 31.9 | 6.341 | **<.0001** |
| II,Male | SS,Male | 0.4533 | 0.0463 | 31.9 | 9.799 | **<.0001** |
| IS,Male | SI,Male | -0.0067 | 0.0463 | 31.9 | -0.144 | >0.9999 |
| IS,Male | SS,Male | 0.1533 | 0.0463 | 31.9 | 3.314 | **0.042** |
| SI,Male | SS,Male | 0.1600 | 0.0463 | 31.9 | 3.459 | **0.0297** |

**Table S2** Summary of the Hybrid Experiment results A) Estimates and 95 % confidence intervals (CIs) for relative hazard rates (ie. the exponent of the coefficients) corresponding to various fixed parameters of the Cox’s proportional hazards model. Hazard rates are expressed relative to the default level of that fixed factor. The default level for “cross” is II, while the default level for “sex” is female. Hazard rates significantly greater than 1 correspond to poorer survivorship relative to the default level. B) An ANOVA-like analysis of deviance table for the logistic regression.

| **A) Hazard Rates for the Cox Proportional Model** | | | |
| --- | --- | --- | --- |
|  | **Hazard Rate** | **Lower CI** | **Upper CI** |
| CrossIS | **2.2798** | **1.7988** | **2.8894** |
| CrossSI | **2.2035** | **1.7374** | **2.7948** |
| CrossSS | **5.4946** | **4.4158** | **6.8369** |
| SexMale | **1.5137** | **1.1264** | **2.0342** |
| CrossIS:SexMale | **1.4706** | **1.0784** | **2.0054** |
| CrossSI:SexMale | **1.4405** | **1.0555** | **1.9659** |
| CrossSS:SexMale | 0.9928 | 0.7417 | 1.3288 |
|  | | | |
| **B) Analysis of Deviance Table for Logistic Regression** | | | |
|  | **Chisq** | **Df** | **Pr(>Chisq)** |
| Cross | 296.51 | 3 | **<0.0001** |
| Sex | 78.306 | 1 | **<0.0001** |
| Cross:Sex | 19.303 | 3 | **0.0002** |

**Table S3** Summary of the X-cloning Experiment results A) Estimates and 95 % confidence intervals (CIs) for relative hazard rates (ie. the exponent of the coefficients) corresponding to various fixed parameters of the Cox’s proportional hazards model. Hazard rates are expressed relative to the default level of that fixed factor. The default level for “Selection Regime” is I, while the default level for “sex” is female. Hazard rates significantly greater than 1 correspond to poorer survivorship relative to the default level. B) An ANOVA-like analysis of deviance table for the logistic regression.

| **A) Hazard Rates for the Cox's Proportional Model** | | | |
| --- | --- | --- | --- |
|  | **Hazard Rate** | **Lower CI** | **Upper CI** |
| Selection.RegimeS | 1.0601 | 0.9503 | 1.1825 |
| SexMale | 1.0131 | 0.9474 | 1.0835 |
| Selection.RegimeS:SexMale | 1.0297 | 0.9368 | 1.1319 |
|  | | | |
| **B) Analysis of Deviance Table for Logistic Regression** | | | |
|  | **Chisq** | **Df** | **Pr(>Chisq)** |
| Selection.Regime | 1.2546 | 1 | 0.2627 |
| Sex | 24.2227 | 1 | **<0.0001** |
| Selection.Regime:Sex | 0.5271 | 1 | 0.4678 |

**Table S4**Deviance Information Criteria (DIC) for the MCMCglmm models for the X-cloning Experiment to test for the effect of X-line. Null models represent models that lack a term corresponding to X-line, while full models represent models that incorporate a random term corresponding to X-line

|  | **Deviance Information Criteria (DIC)** | |
| --- | --- | --- |
| **Selection Regime** | **Null Model** | **Full Model** |
| **I** | 1342.306 | 1344.905 |
| **S** | 1351.837 | 1354.273 |

| **Table S5A.**Results of correlation analysis between median time to death of males and females of the same X line by i) Linear Model and ii) Spearman’s rank correlation | | | | | |
| --- | --- | --- | --- | --- | --- |
| **i) Linear Model** | | | | | |
|  |  | Estimate | Std. Error | t value | p value |
| I1 | (Intercept) | 1219.7436 | 151.8339 | 8.0330 | **<0.0001** |
|  | Male | -0.1162 | 0.1281 | -0.9070 | 0.3770 |
| I2 | (Intercept) | 561.9780 | 164.5289 | 3.4160 | **0.0031** |
|  | Male | 0.2787 | 0.2094 | 1.3310 | 0.1998 |
| I3 | (Intercept) | 1554.2376 | 584.9196 | 2.6570 | **0.0160** |
|  | Male | -0.5193 | 0.6858 | -0.7570 | 0.4590 |
| I4 | (Intercept) | 851.7619 | 66.0325 | 12.8990 | **<0.0001** |
|  | Male | -0.0556 | 0.0558 | -0.9970 | 0.3320 |
| S1 | (Intercept) | 1150.9281 | 213.7533 | 5.3840 | **<0.0001** |
|  | Male | -0.0835 | 0.2059 | -0.4050 | 0.6900 |
| S2 | (Intercept) | 789.7930 | 368.2072 | 2.1450 | **0.0459** |
|  | Male | 0.0504 | 0.5246 | 0.0960 | 0.9245 |
| S3 | (Intercept) | 860.8082 | 154.4088 | 5.5750 | **<0.0001** |
|  | Male | 0.0413 | 0.1325 | 0.3120 | 0.7590 |
| S4 | (Intercept) | 821.3178 | 135.2469 | 6.0730 | **<0.0001** |
|  | Male | -0.0336 | 0.1568 | -0.2140 | 0.8330 |
| **ii) Spearman's Rank Correlation** | | | | | |
|  | S | Rho | p value |  |  |
| I1 | 1142.5 | -0.0022 | 0.9929 |  |  |
| I2 | 722.04 | 0.4571 | **0.0427** |  |  |
| I3 | 1430.9 | -0.0758 | 0.7506 |  |  |
| I4 | 1643.7 | -0.2358 | 0.3168 |  |  |
| S1 | 1255 | 0.0564 | 0.8132 |  |  |
| S2 | 987.65 | 0.2574 | 0.2732 |  |  |
| S3 | 1098.8 | 0.1738 | 0.4636 |  |  |
| S4 | 1321.9 | 0.0061 | 0.9798 |  |  |

| **Table S5B.**Results of correlation analysis between proportion survivorship of males and females of the same X line by i) Linear Model and ii) Spearman’s rank correlation | | | | | |
| --- | --- | --- | --- | --- | --- |
| - 1. **Linear Model** | | | | | |
|  |  | Estimate | Std. Error | t value | p value |
| I1 | (Intercept) | 0.0601 | 0.0195 | 3.0730 | **0.0069** |
|  | Male | 0.0638 | 0.1564 | 0.4080 | 0.6884 |
| I2 | (Intercept) | 0.0253 | 0.0114 | 2.2140 | **0.0400** |
|  | Male | 0.4529 | 0.1446 | 3.1330 | **0.0058** |
| I3 | (Intercept) | 0.0898 | 0.0277 | 3.2470 | **0.0045** |
|  | Male | -0.5081 | 0.4261 | -1.1920 | 0.2486 |
| I4 | (Intercept) | 0.0046 | 0.0067 | 0.6880 | **0.5000** |
|  | Male | 0.0718 | 0.0537 | 1.3370 | 0.1980 |
| S1 | (Intercept) | 0.0445 | 0.0163 | 2.7360 | **0.0136** |
|  | Male | -0.0039 | 0.1509 | -0.0260 | 0.9797 |
| S2 | (Intercept) | 0.0491 | 0.0100 | 4.9080 | **0.0001** |
|  | Male | -0.1965 | 0.2063 | -0.9530 | 0.3533 |
| S3 | (Intercept) | 0.0481 | 0.0179 | 2.6810 | **0.0153** |
|  | Male | -0.0925 | 0.1620 | -0.5710 | 0.5752 |
| S4 | (Intercept) | 0.0136 | 0.0073 | 1.8540 | 0.0802 |
|  | Male | 0.0157 | 0.0903 | 0.1730 |  |
| **ii) Spearman's Rank Correlation** | | | | | |
|  | S | Rho | p value |  |  |
| I1 | 934.09 | 0.1806 | 0.4593 |  |  |
| I2 | 810.33 | 0.3907 | 0.0885 |  |  |
| I3 | 1486.8 | -0.1179 | 0.6206 |  |  |
| I4 | 932.02 | 0.2992 | 0.2000 |  |  |
| S1 | 1232.9 | 0.0730 | 0.7597 |  |  |
| S2 | 1657.3 | -0.2461 | 0.2955 |  |  |
| S3 | 1463.5 | -0.1004 | 0.6736 |  |  |
| S4 | 1441.4 | -0.0838 | 0.7254 |  |  |
